# Supplementary material for: Multiple cullin-associated E3 ligases regulate cyclin D1 protein stability
Source: eLife. 2023 Nov 9;12:e80327. doi: 10.7554/eLife.80327 (PMC10651173; doi:10.7554/eLife.80327)
Supplement: Figure 1—source data 2. [file elife-80327-fig1-data2.zip › Original western blot files for Figure 1.pdf]

Figure 1A

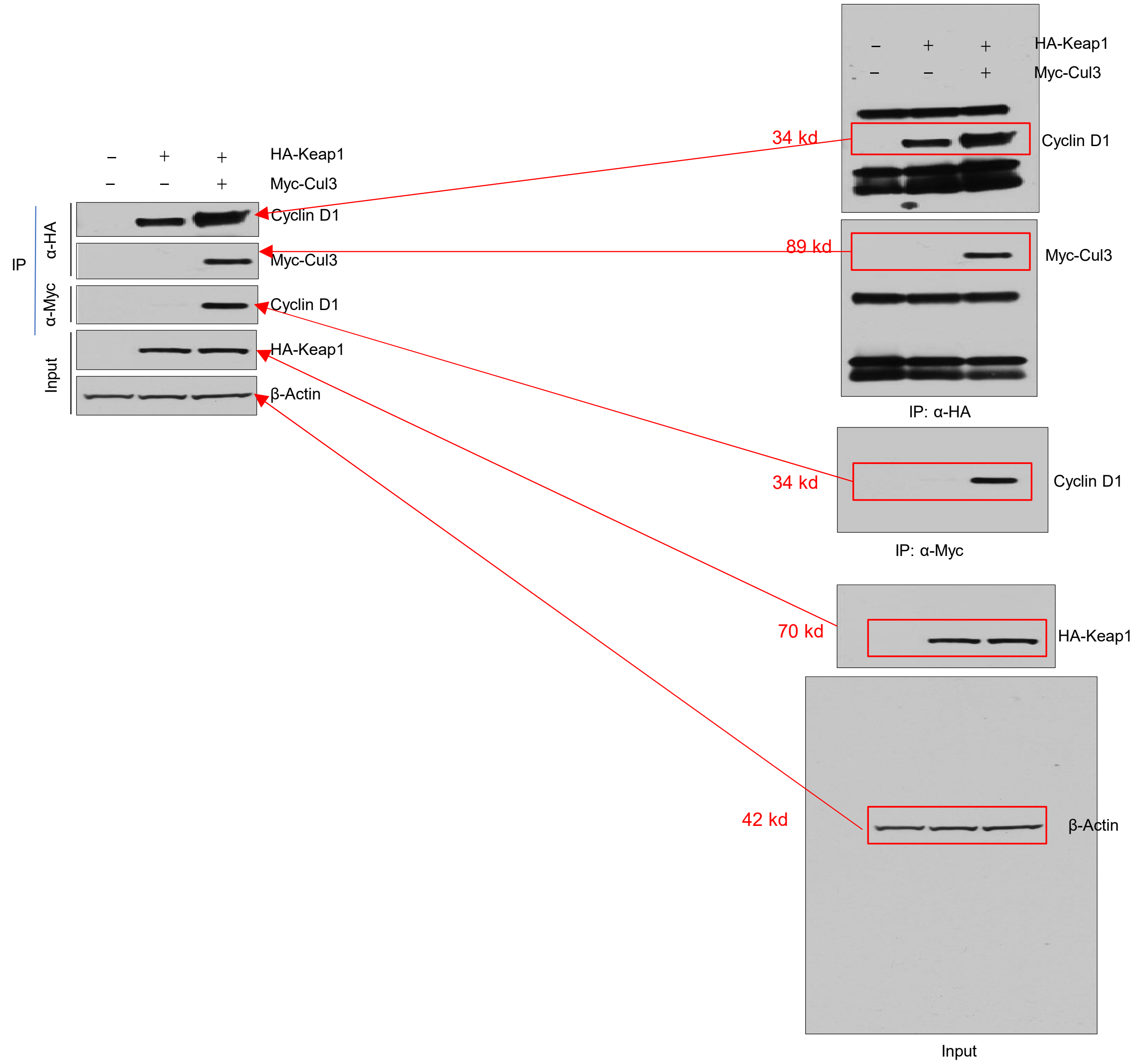

–
+
+
HA-Keap1
–
–
+
Myc-Cul3
IP
α-HA
α-Myc
Input
Cyclin D1
Myc-Cul3
Cyclin D1
HA-Keap1
β-Actin
34 kd
89 kd
34 kd
70 kd
42 kd
IP: α-HA
IP: α-Myc
Input

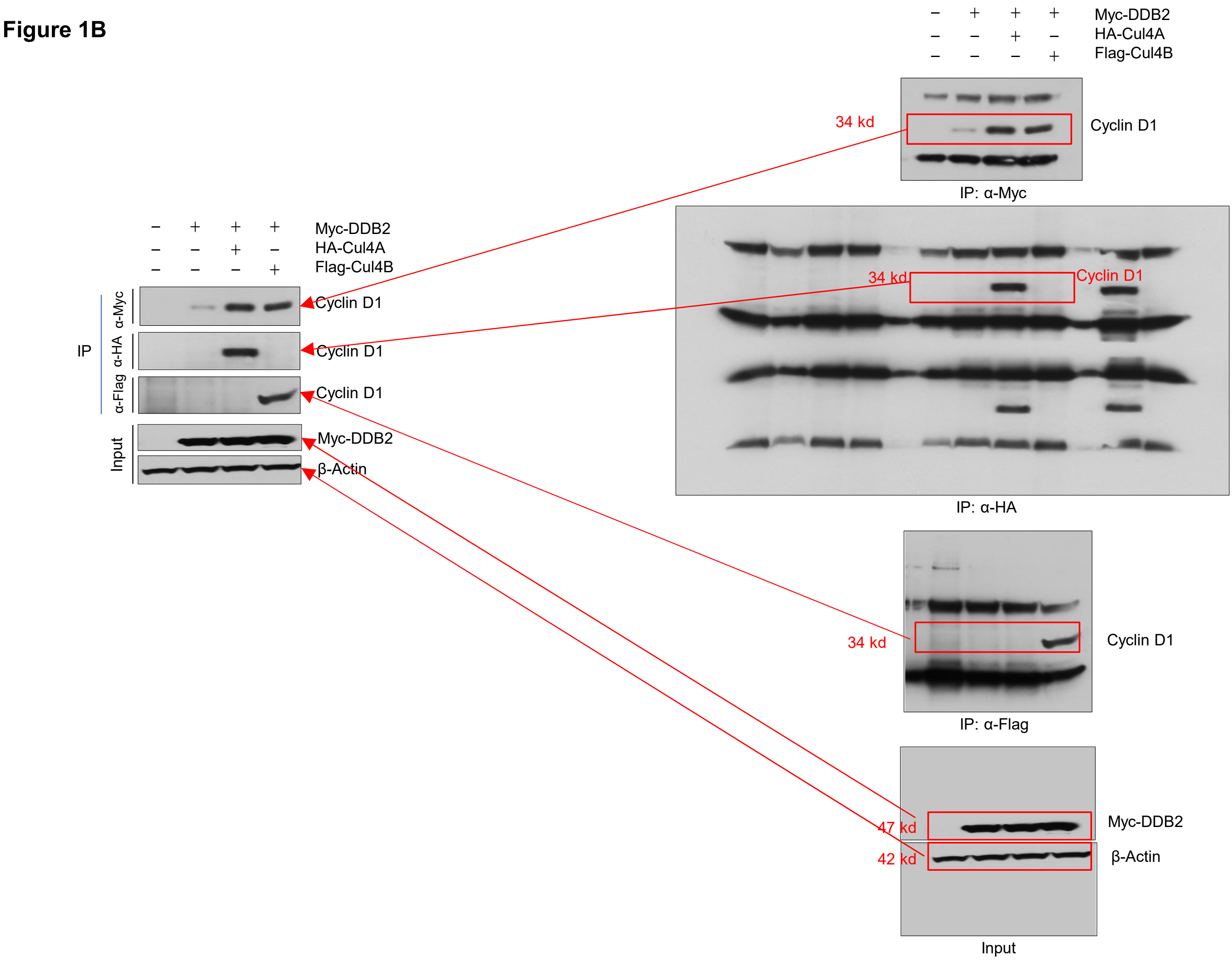
Figure 1B
Myc-DDB2
HA-Cul4A
Flag-Cul4B
34 kd
Cyclin D1
IP: α-Myc
IP: α-HA
IP: α-Flag
47 kd
42 kd
Myc-DDB2
β-Actin
Input
IP
α-Myc
α-HA
α-Flag

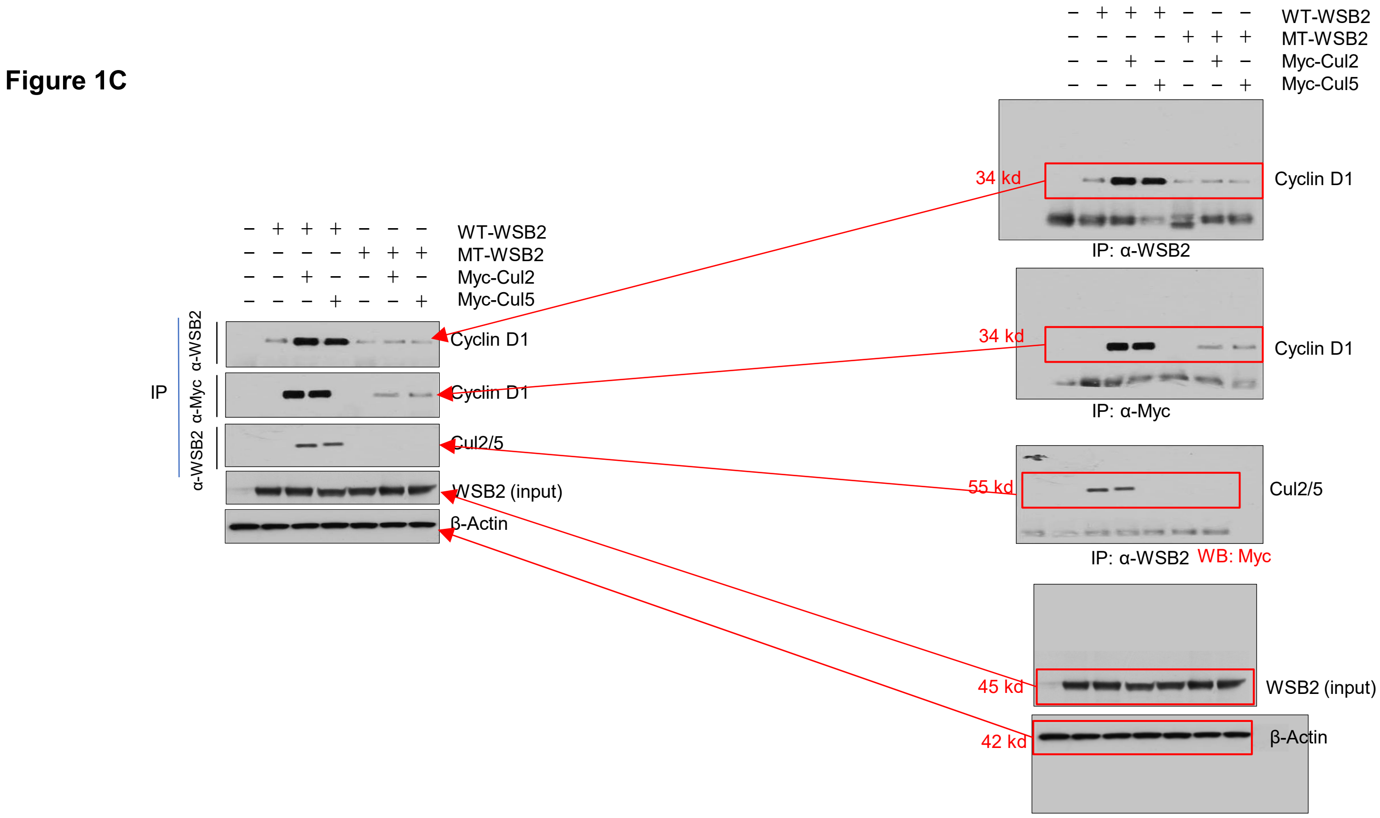
Figure 1C
WT-WSB2
MT-WSB2
Myc-Cul2
Myc-Cul5
34 kd
Cyclin D1
IP: α-WSB2
IP: α-Myc
55 kd
Cul2/5
IP: α-WSB2 WB: Myc
45 kd
WSB2 (input)
42 kd
β-Actin
IP
α-WSB2
α-Myc

Figure 1D

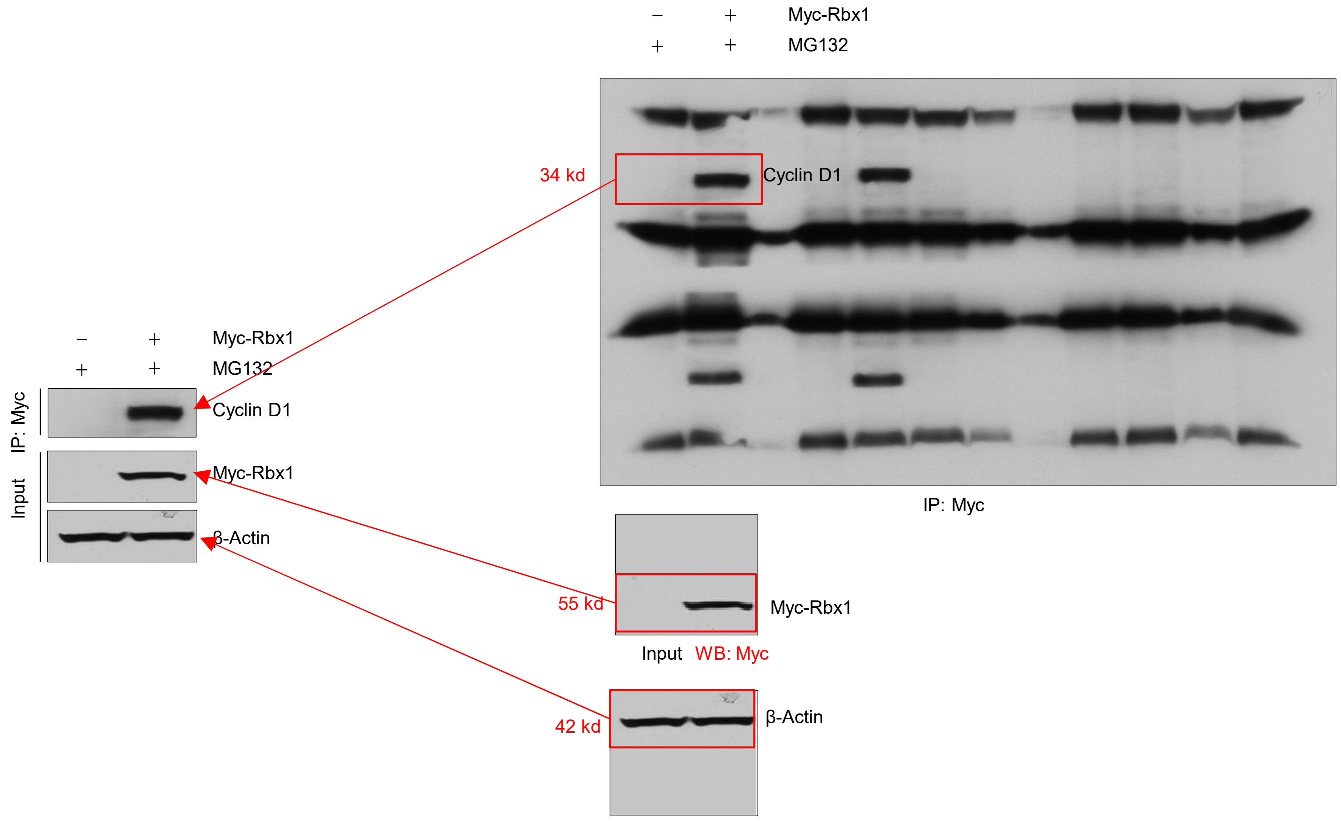

Figure 1E

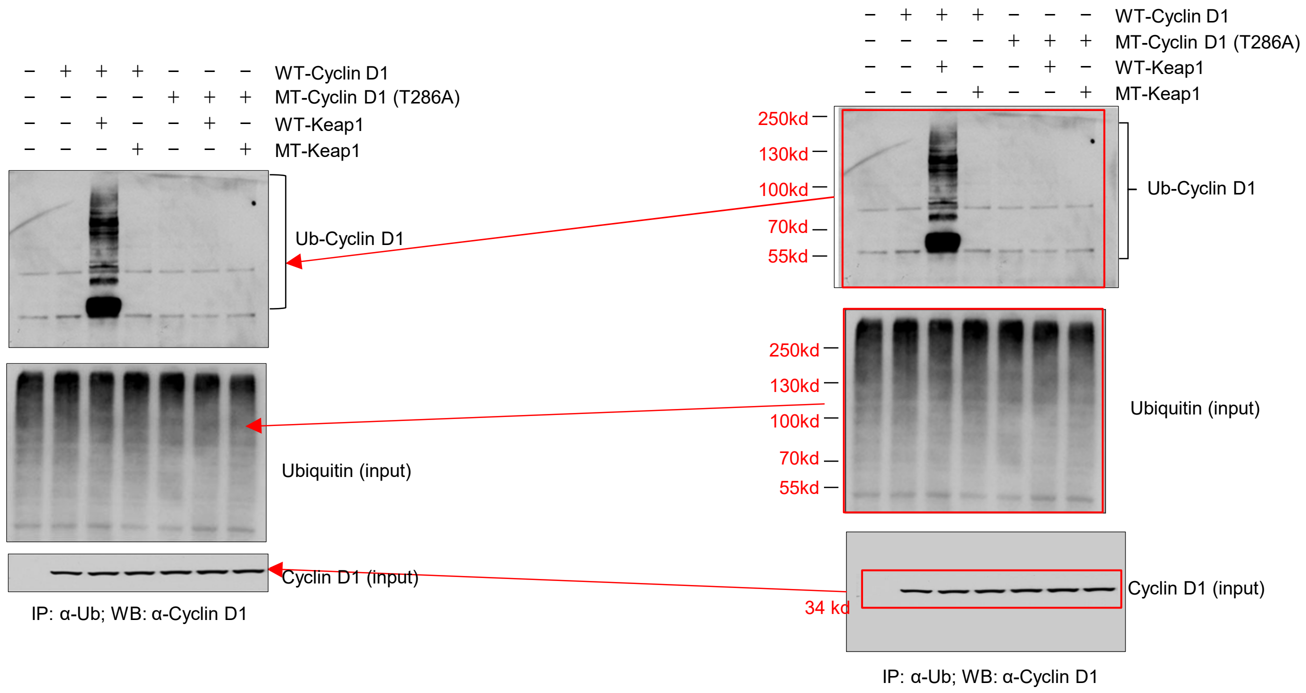

Figure 1F

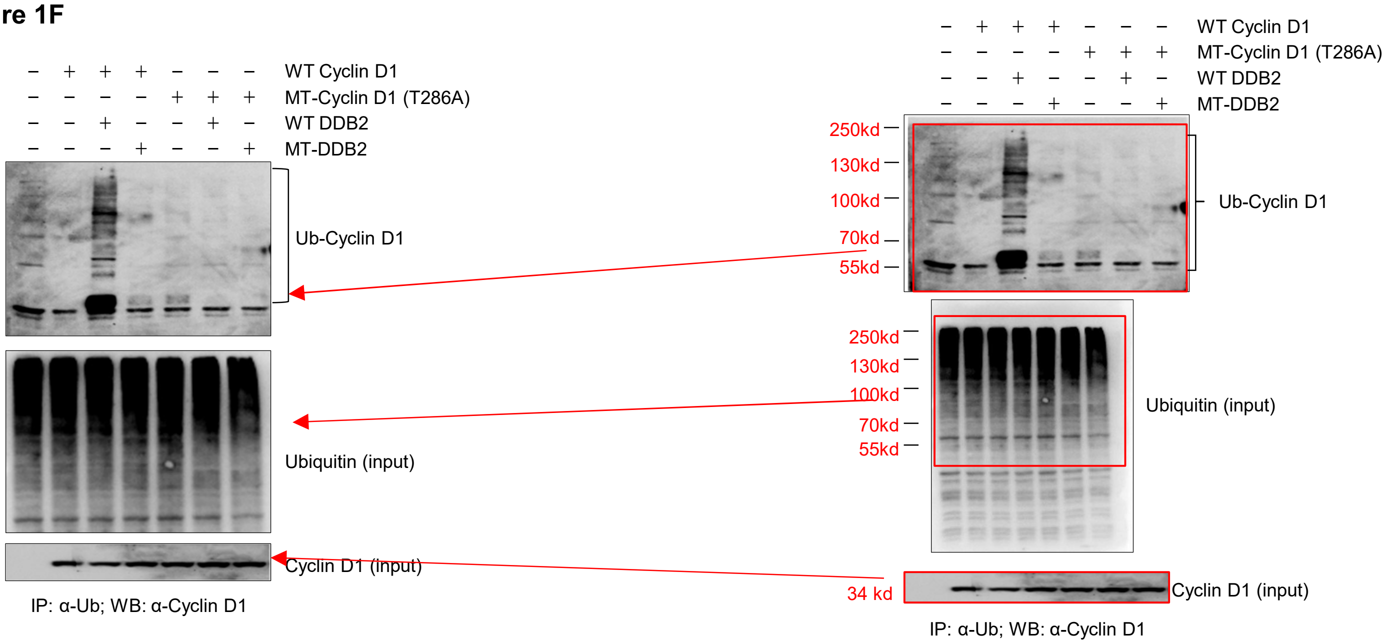

Figure 1G

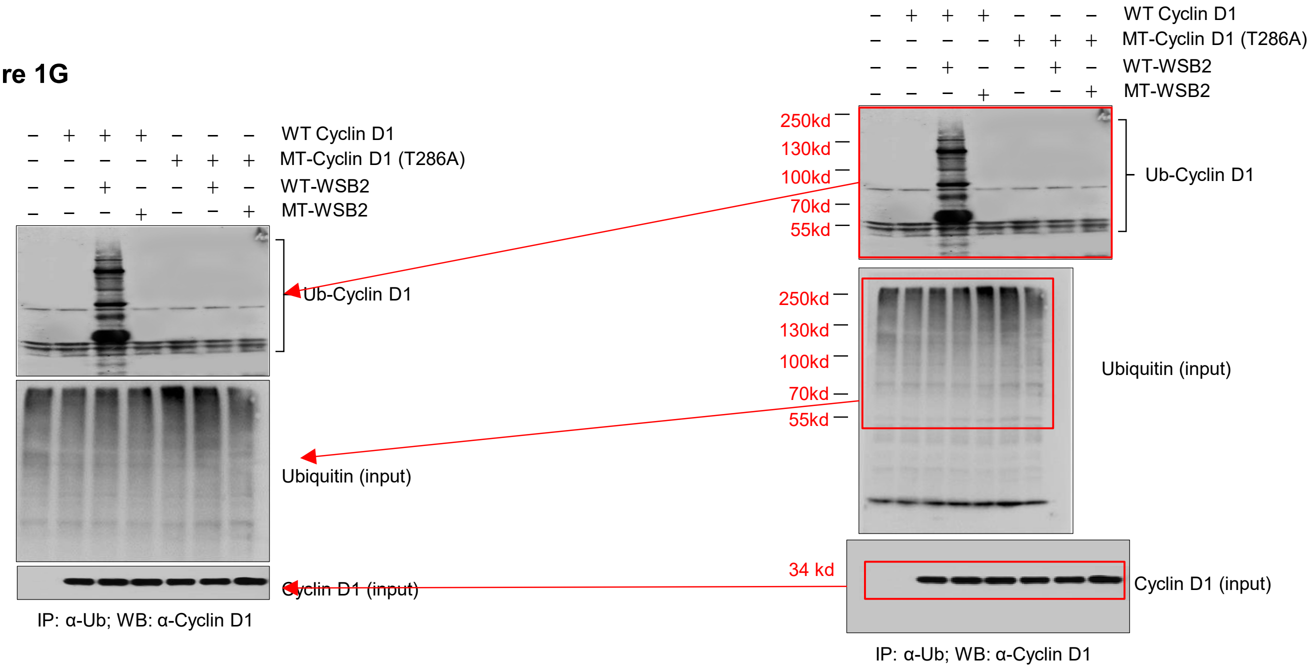

Figure 1H

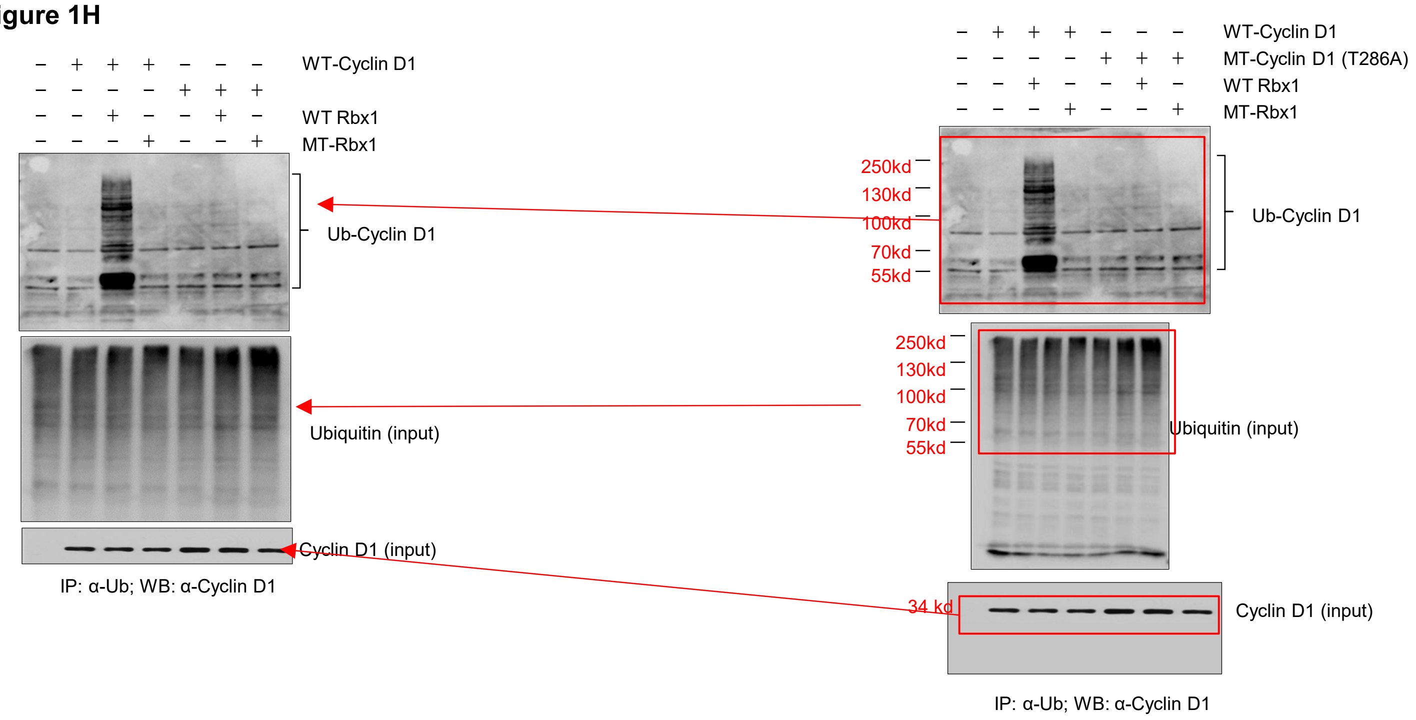

Figure 1I

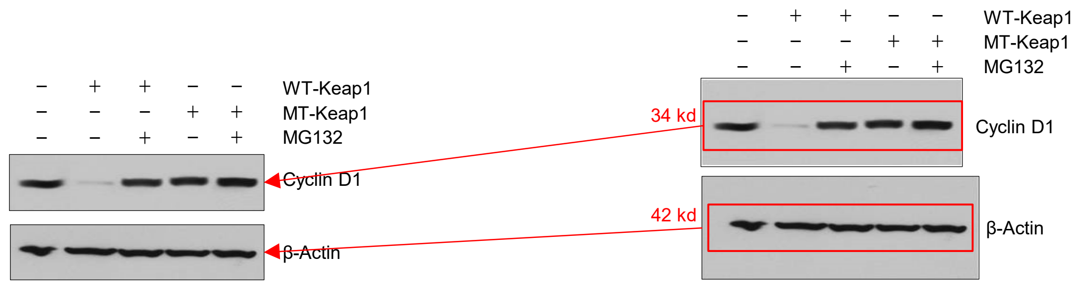

Figure 1J

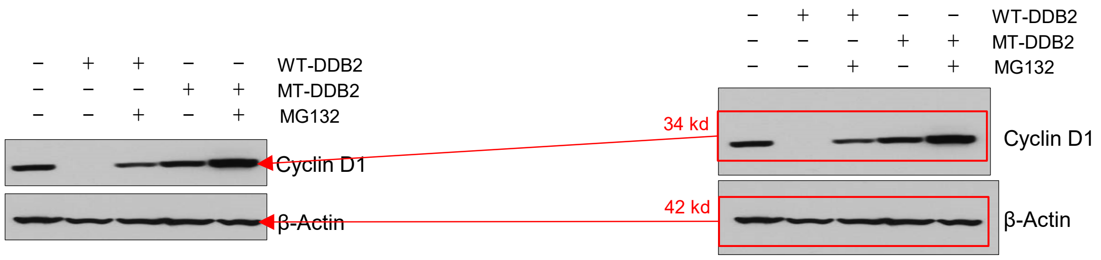

Figure 1K

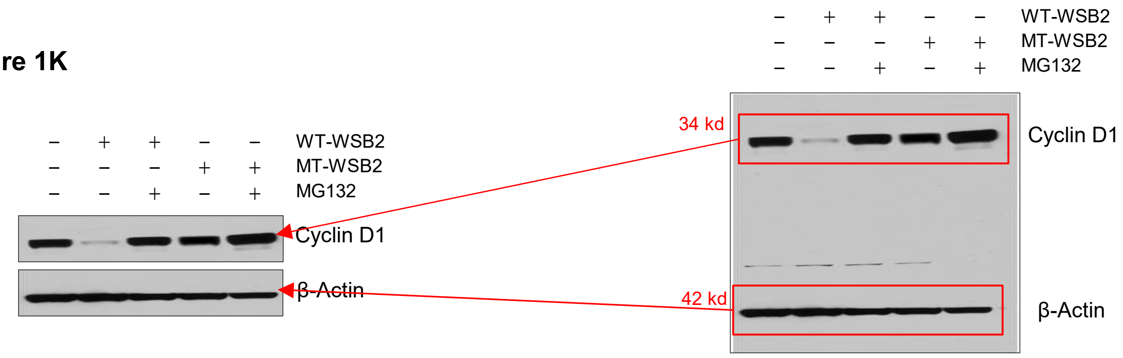

Figure 1L

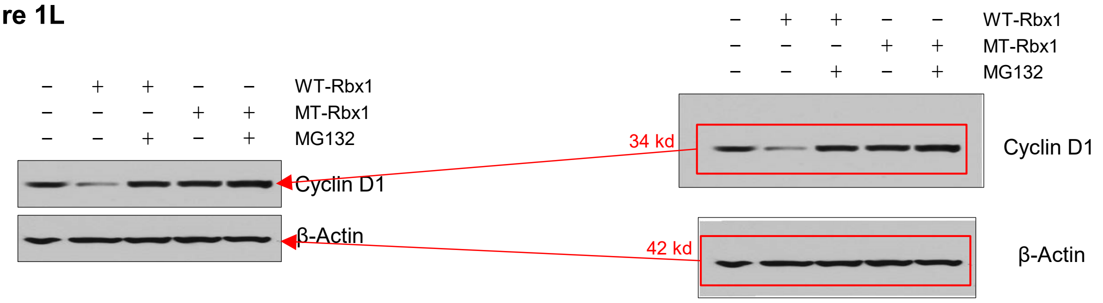

Figure 1M

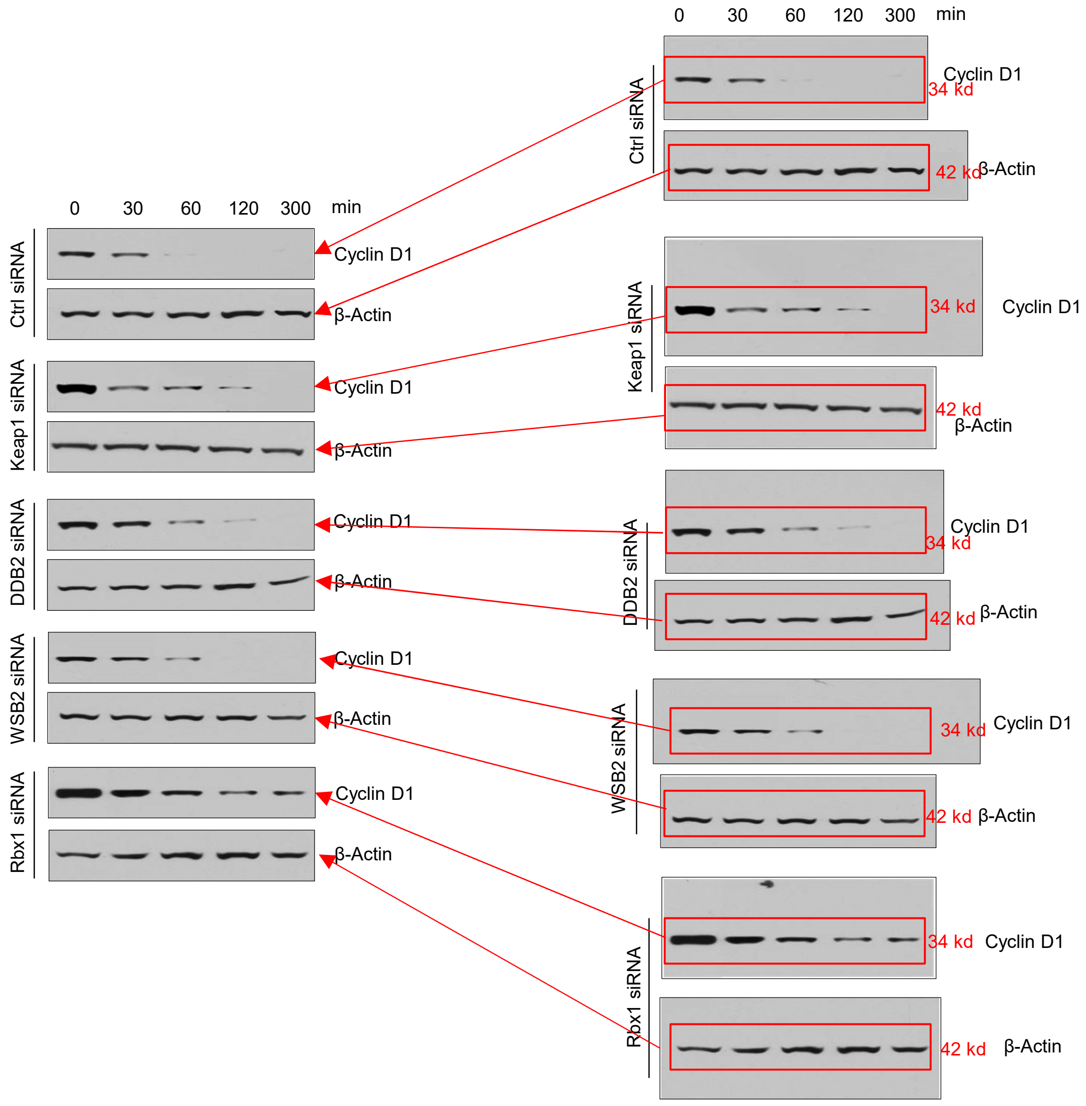

0 30 60 120 300 min
Ctrl siRNA
Keap1 siRNA
DDB2 siRNA
WSB2 siRNA
Rbx1 siRNA
Cyclin D1
β-Actin
34 kd
42 kd
