# Supplementary figures and images for: Multiple cullin-associated E3 ligases regulate cyclin D1 protein stability
Source: eLife. 2023 Nov 9;12:e80327. doi: 10.7554/eLife.80327 (PMC10651173; doi:10.7554/eLife.80327)

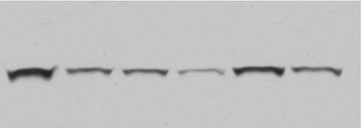

Supplement: Figure 1—source data 2. [file elife-80327-fig1-data2.zip › Figure1-Figure supplement 11-1.jpg]

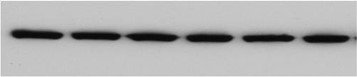

Supplement: Figure 1—source data 2. [file elife-80327-fig1-data2.zip › Figure1-Figure supplement 11-2.jpg]

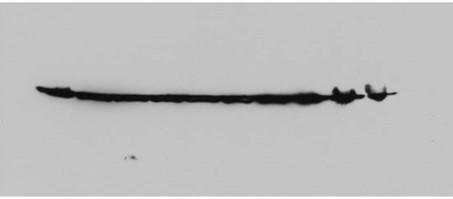

Supplement: Figure 1—source data 2. [file elife-80327-fig1-data2.zip › Figure1-Figure supplement 11-3.jpg]

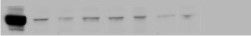

Supplement: Figure 1—source data 2. [file elife-80327-fig1-data2.zip › Figure1-Figure supplement 1A-1.jpg]

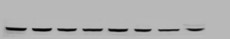

Supplement: Figure 1—source data 2. [file elife-80327-fig1-data2.zip › Figure1-Figure supplement 1A-2.jpg]

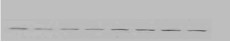

Supplement: Figure 1—source data 2. [file elife-80327-fig1-data2.zip › Figure1-Figure supplement 1A-3.jpg]

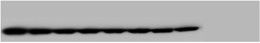

Supplement: Figure 1—source data 2. [file elife-80327-fig1-data2.zip › Figure1-Figure supplement 1A-4.jpg]

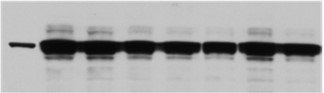

Supplement: Figure 1—source data 2. [file elife-80327-fig1-data2.zip › Figure1-Figure supplement 1B-1.jpg]

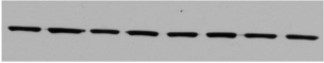

Supplement: Figure 1—source data 2. [file elife-80327-fig1-data2.zip › Figure1-Figure supplement 1B-2.jpg]

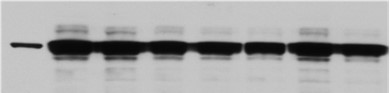

Supplement: Figure 1—source data 2. [file elife-80327-fig1-data2.zip › Figure1-Figure supplement 3-1.jpg]

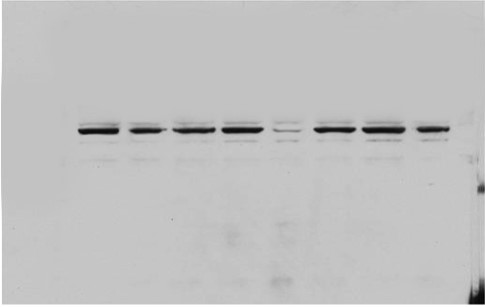

Supplement: Figure 1—source data 2. [file elife-80327-fig1-data2.zip › Figure1-Figure supplement 3-2.jpg]

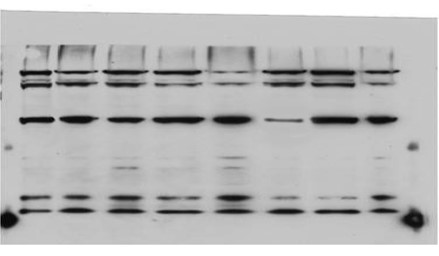

Supplement: Figure 1—source data 2. [file elife-80327-fig1-data2.zip › Figure1-Figure supplement 3-3.jpg]

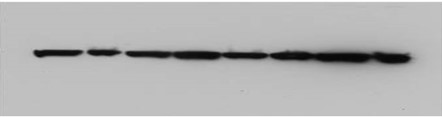

Supplement: Figure 1—source data 2. [file elife-80327-fig1-data2.zip › Figure1-Figure supplement 3-4.jpg]

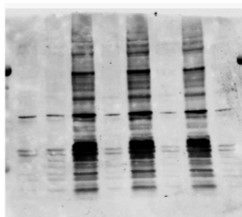

Supplement: Figure 1—source data 2. [file elife-80327-fig1-data2.zip › Figure1-Figure supplement 7-1.jpg]

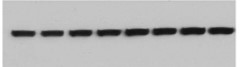

Supplement: Figure 1—source data 2. [file elife-80327-fig1-data2.zip › Figure1-Figure supplement 7-2.jpg]

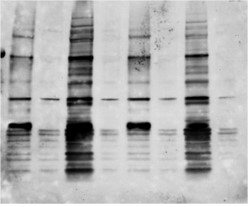

Supplement: Figure 1—source data 2. [file elife-80327-fig1-data2.zip › Figure1-Figure supplement 7-3.jpg]

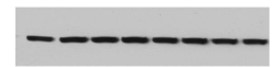

Supplement: Figure 1—source data 2. [file elife-80327-fig1-data2.zip › Figure1-Figure supplement 7-4.jpg]

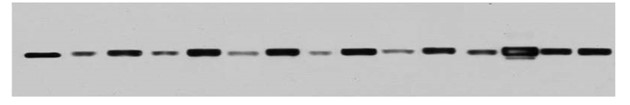

Supplement: Figure 1—source data 2. [file elife-80327-fig1-data2.zip › Figure1-Figure supplement 8-1.jpg]

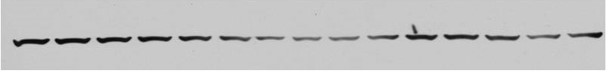

Supplement: Figure 1—source data 2. [file elife-80327-fig1-data2.zip › Figure1-Figure supplement 8-2.jpg]

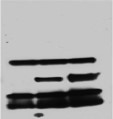

Supplement: Figure 1—source data 2. [file elife-80327-fig1-data2.zip › Figure1A-1.jpg]

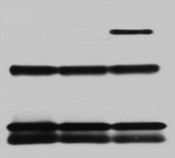

Supplement: Figure 1—source data 2. [file elife-80327-fig1-data2.zip › Figure1A-2.jpg]

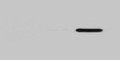

Supplement: Figure 1—source data 2. [file elife-80327-fig1-data2.zip › Figure1A-3.jpg]

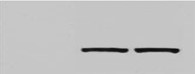

Supplement: Figure 1—source data 2. [file elife-80327-fig1-data2.zip › Figure1A-4.jpg]

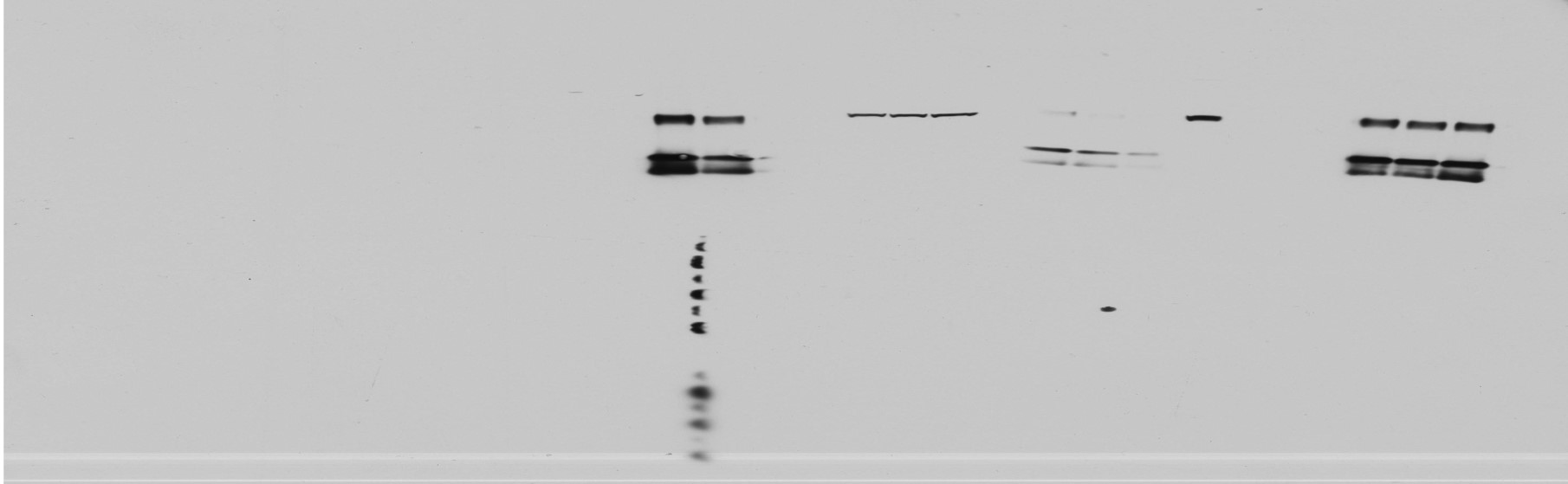

Supplement: Figure 1—source data 2. [file elife-80327-fig1-data2.zip › Figure1A-5.jpg]

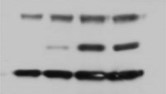

Supplement: Figure 1—source data 2. [file elife-80327-fig1-data2.zip › Figure1B-1.jpg]

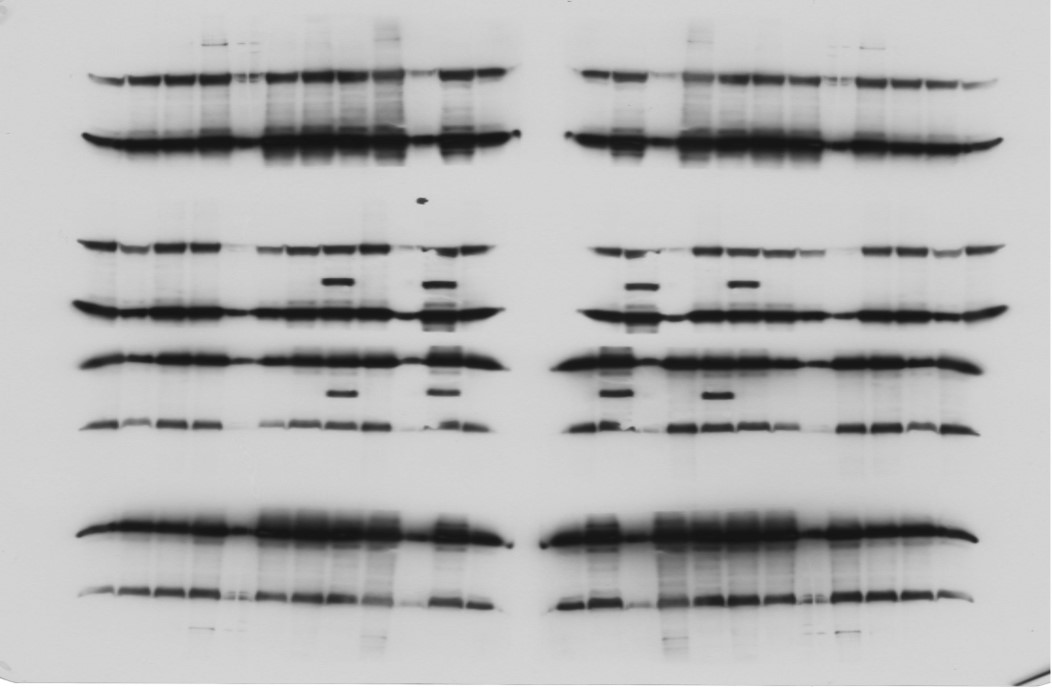

Supplement: Figure 1—source data 2. [file elife-80327-fig1-data2.zip › Figure1B-2.jpg]

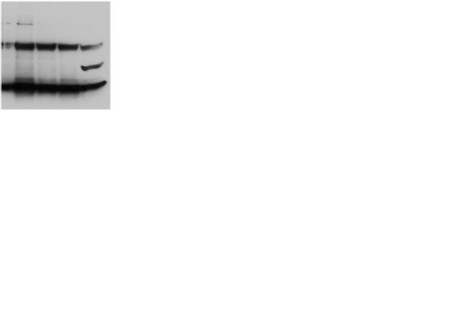

Supplement: Figure 1—source data 2. [file elife-80327-fig1-data2.zip › Figure1B-3.jpg]

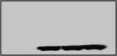

Supplement: Figure 1—source data 2. [file elife-80327-fig1-data2.zip › Figure1B-4.jpg]

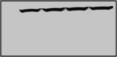

Supplement: Figure 1—source data 2. [file elife-80327-fig1-data2.zip › Figure1B-5.jpg]

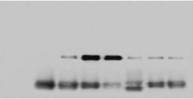

Supplement: Figure 1—source data 2. [file elife-80327-fig1-data2.zip › Figure1C-1.jpg]

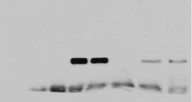

Supplement: Figure 1—source data 2. [file elife-80327-fig1-data2.zip › Figure1C-2.jpg]

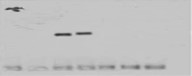

Supplement: Figure 1—source data 2. [file elife-80327-fig1-data2.zip › Figure1C-3.jpg]

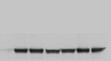

Supplement: Figure 1—source data 2. [file elife-80327-fig1-data2.zip › Figure1C-4.jpg]

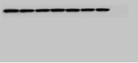

Supplement: Figure 1—source data 2. [file elife-80327-fig1-data2.zip › Figure1C-5.jpg]

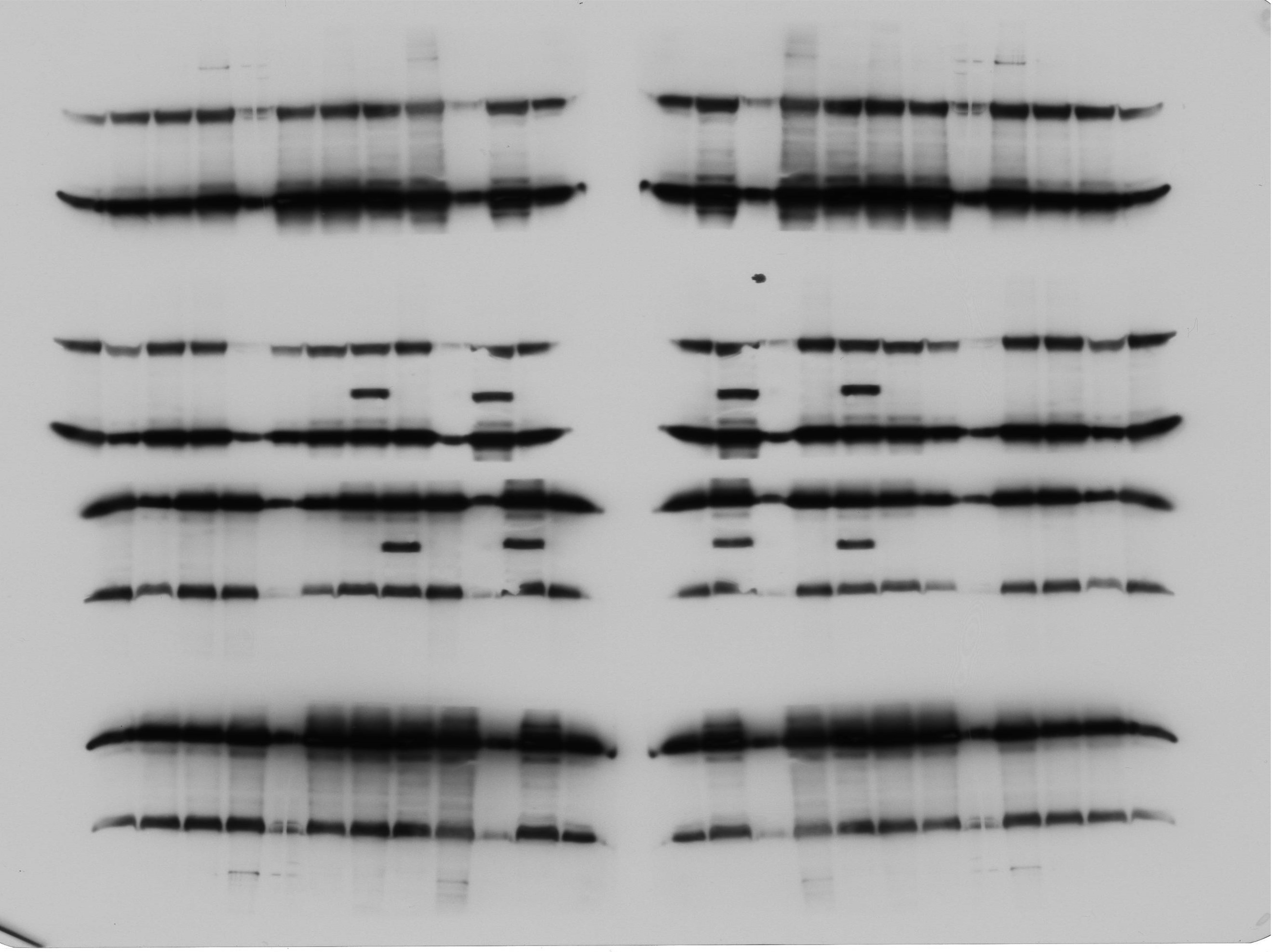

Supplement: Figure 1—source data 2. [file elife-80327-fig1-data2.zip › Figure1D-1.jpg]

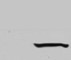

Supplement: Figure 1—source data 2. [file elife-80327-fig1-data2.zip › Figure1D-2.jpg]

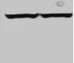

Supplement: Figure 1—source data 2. [file elife-80327-fig1-data2.zip › Figure1D-3.jpg]

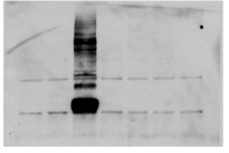

Supplement: Figure 1—source data 2. [file elife-80327-fig1-data2.zip › Figure1E-1.jpg]

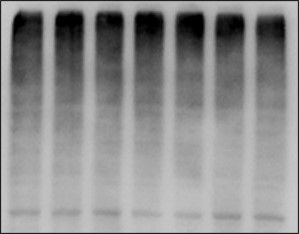

Supplement: Figure 1—source data 2. [file elife-80327-fig1-data2.zip › Figure1E-2.jpg]

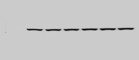

Supplement: Figure 1—source data 2. [file elife-80327-fig1-data2.zip › Figure1E-3.jpg]

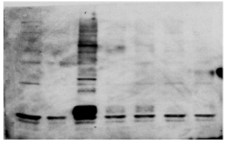

Supplement: Figure 1—source data 2. [file elife-80327-fig1-data2.zip › Figure1F-1.jpg]

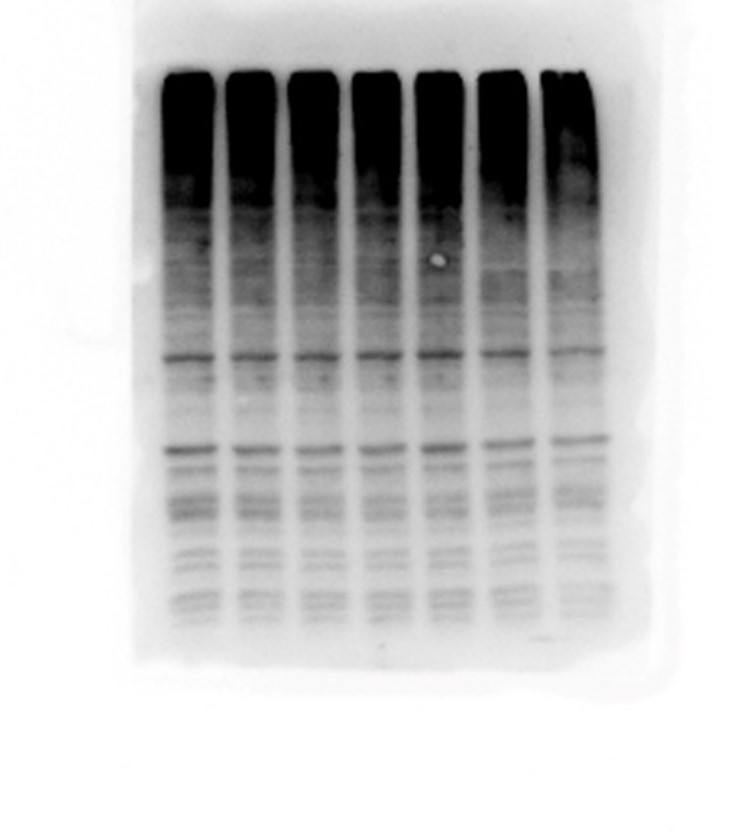

Supplement: Figure 1—source data 2. [file elife-80327-fig1-data2.zip › Figure1F-2.jpg]

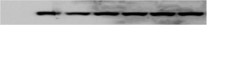

Supplement: Figure 1—source data 2. [file elife-80327-fig1-data2.zip › Figure1F-3.jpg]

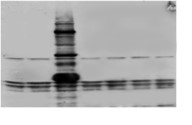

Supplement: Figure 1—source data 2. [file elife-80327-fig1-data2.zip › Figure1G-1.jpg]

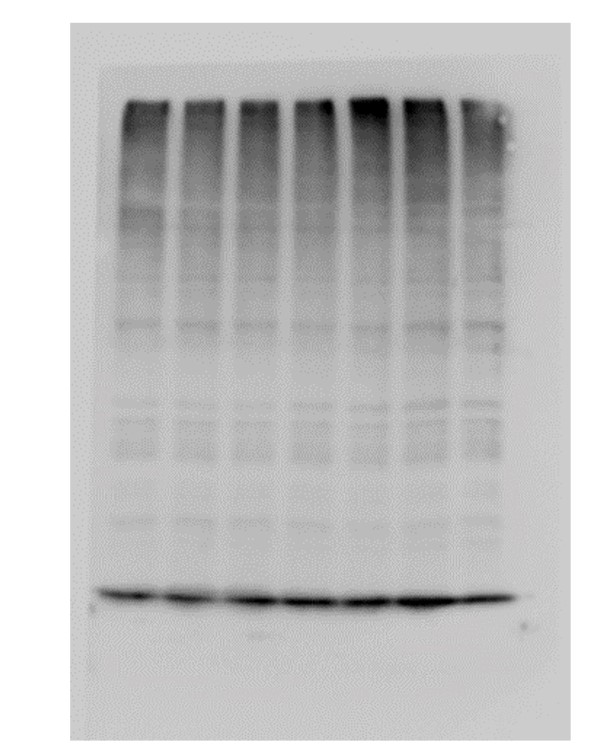

Supplement: Figure 1—source data 2. [file elife-80327-fig1-data2.zip › Figure1G-2.jpg]

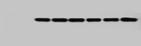

Supplement: Figure 1—source data 2. [file elife-80327-fig1-data2.zip › Figure1G-3.jpg]

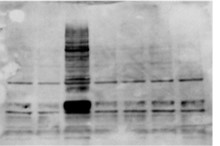

Supplement: Figure 1—source data 2. [file elife-80327-fig1-data2.zip › Figure1H-1.jpg]

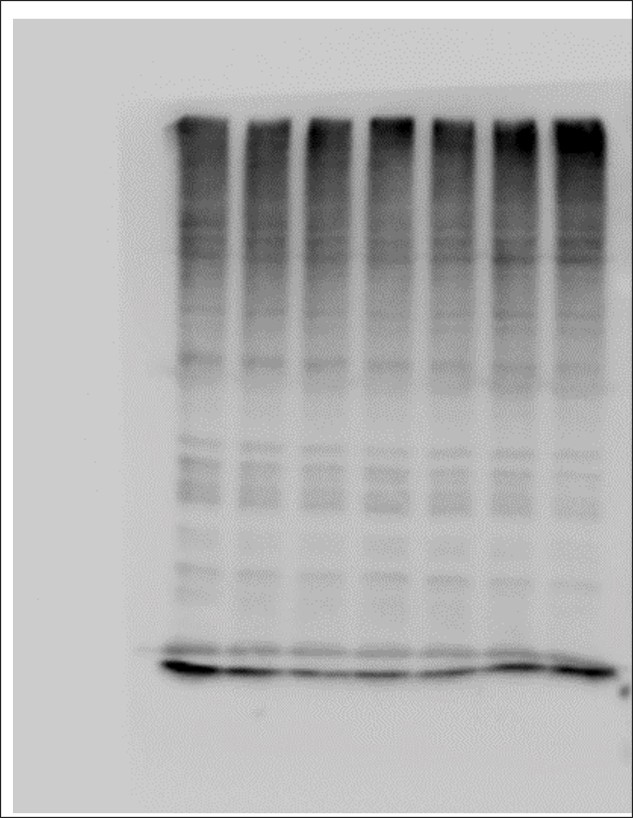

Supplement: Figure 1—source data 2. [file elife-80327-fig1-data2.zip › Figure1H-2.jpg]

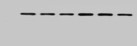

Supplement: Figure 1—source data 2. [file elife-80327-fig1-data2.zip › Figure1H-3.jpg]

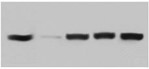

Supplement: Figure 1—source data 2. [file elife-80327-fig1-data2.zip › Figure1I-1.jpg]

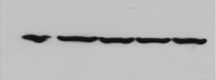

Supplement: Figure 1—source data 2. [file elife-80327-fig1-data2.zip › Figure1I-2.jpg]

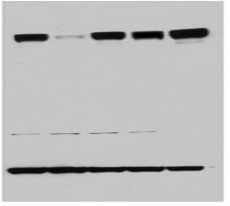

Supplement: Figure 1—source data 2. [file elife-80327-fig1-data2.zip › Figure1K.jpg]

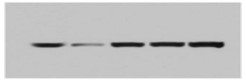

Supplement: Figure 1—source data 2. [file elife-80327-fig1-data2.zip › Figure1L-1.jpg]

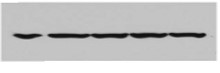

Supplement: Figure 1—source data 2. [file elife-80327-fig1-data2.zip › Figure1L-2.jpg]

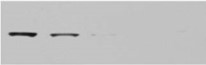

Supplement: Figure 1—source data 2. [file elife-80327-fig1-data2.zip › Figure1M-1.jpg]

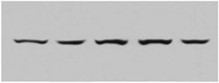

Supplement: Figure 1—source data 2. [file elife-80327-fig1-data2.zip › Figure1M-10.jpg]

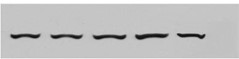

Supplement: Figure 1—source data 2. [file elife-80327-fig1-data2.zip › Figure1M-2.jpg]

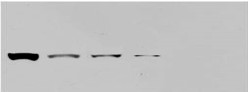

Supplement: Figure 1—source data 2. [file elife-80327-fig1-data2.zip › Figure1M-3.jpg]

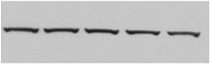

Supplement: Figure 1—source data 2. [file elife-80327-fig1-data2.zip › Figure1M-4.jpg]

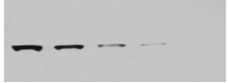

Supplement: Figure 1—source data 2. [file elife-80327-fig1-data2.zip › Figure1M-5.jpg]

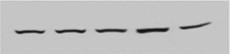

Supplement: Figure 1—source data 2. [file elife-80327-fig1-data2.zip › Figure1M-6.jpg]

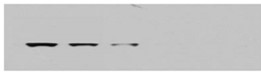

Supplement: Figure 1—source data 2. [file elife-80327-fig1-data2.zip › Figure1M-7.jpg]

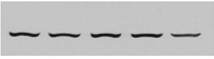

Supplement: Figure 1—source data 2. [file elife-80327-fig1-data2.zip › Figure1M-8.jpg]

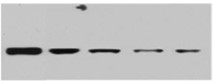

Supplement: Figure 1—source data 2. [file elife-80327-fig1-data2.zip › Figure1M-9.jpg]

**Figure 1-figure supplement 1A**

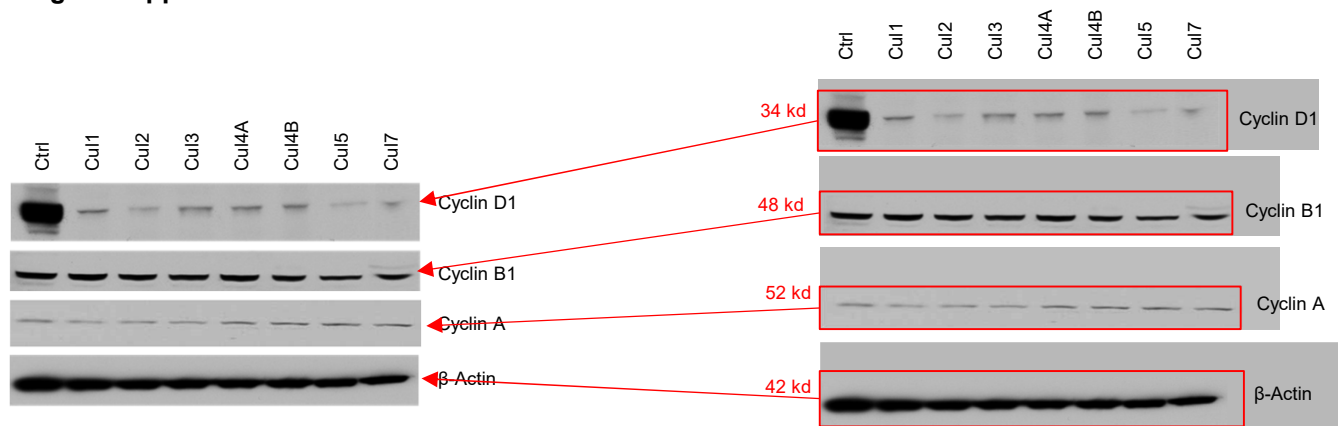

**Figure 1-figure supplement 1B**

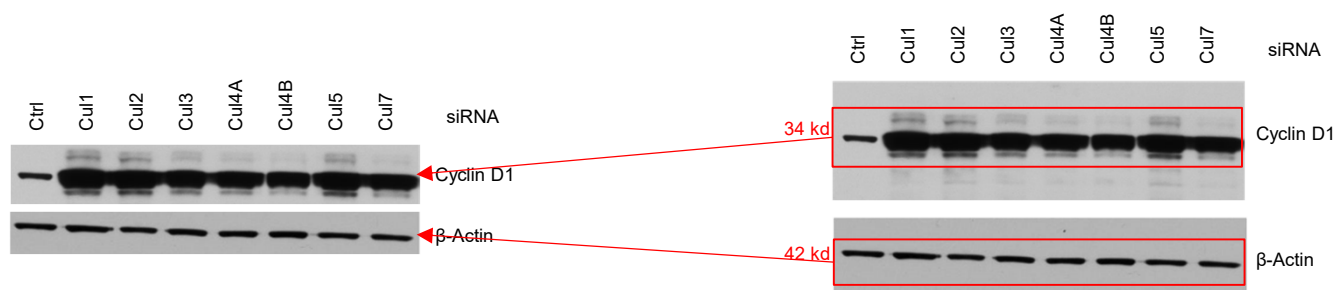

Supplement: Figure 1—source data 2. [file elife-80327-fig1-data2.zip › Original western blot files for Figure 1-figure supplement 1.pdf]

Figure 1-figure supplement 11

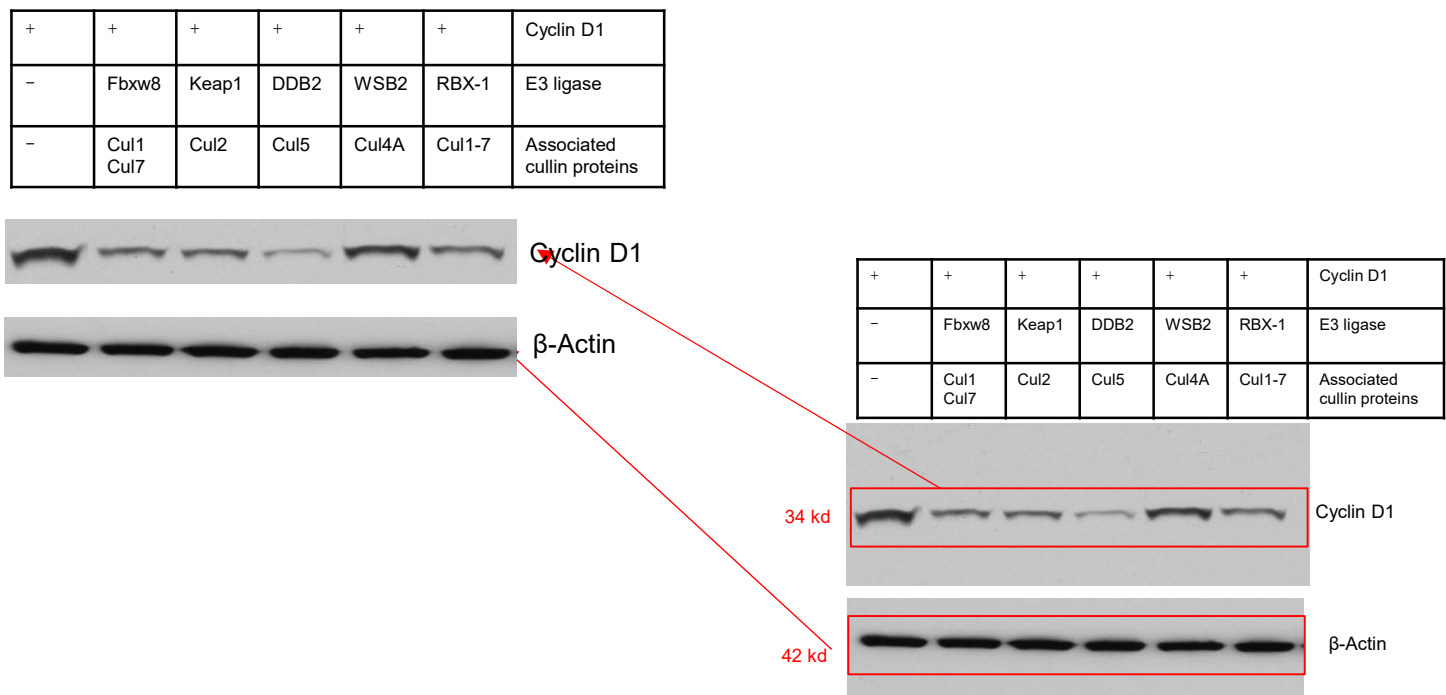

Supplement: Figure 1—source data 2. [file elife-80327-fig1-data2.zip › Original western blot files for Figure 1-figure supplement 11.pdf]

Figure 1-figure supplement 3

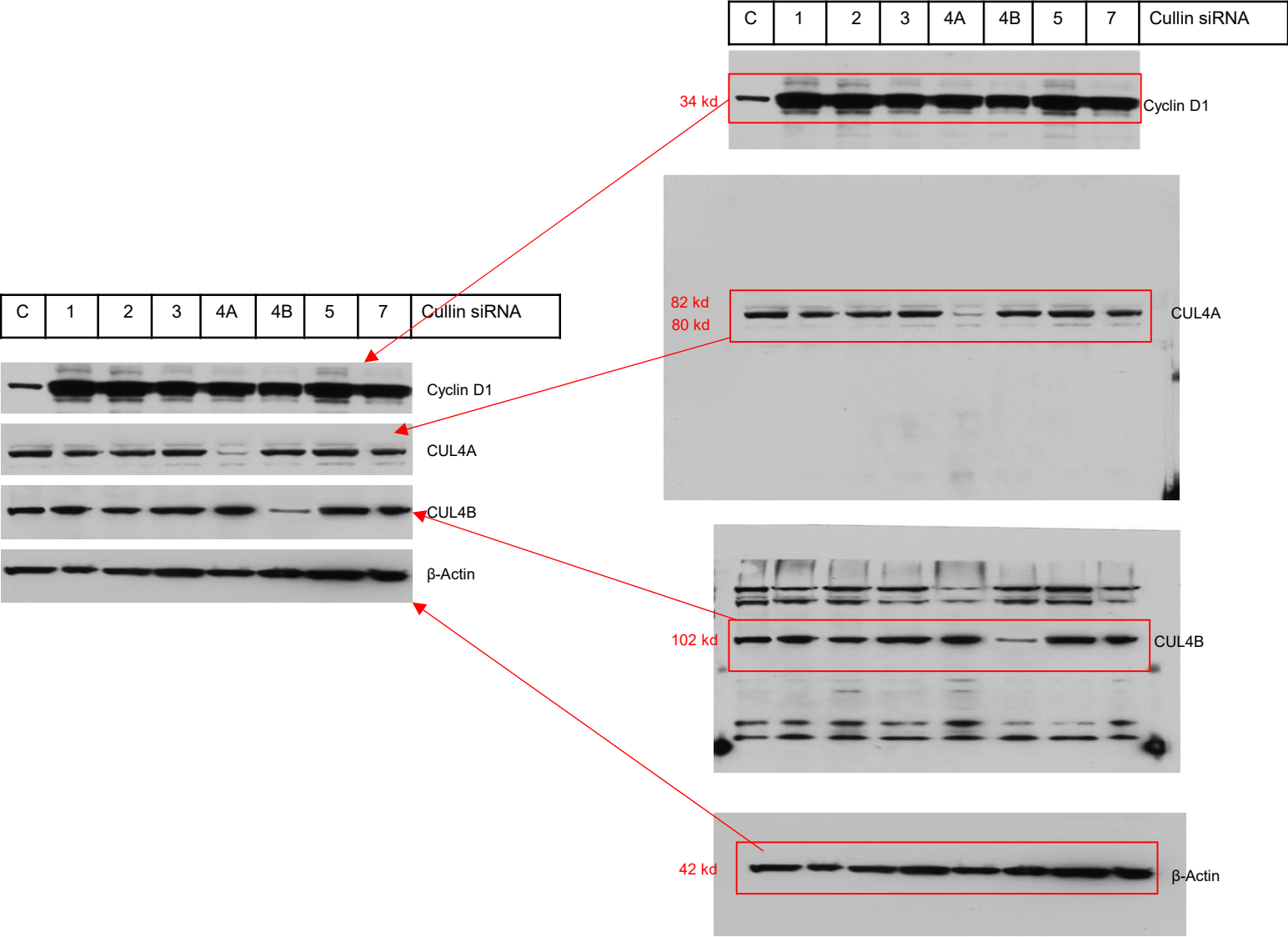

Supplement: Figure 1—source data 2. [file elife-80327-fig1-data2.zip › Original western blot files for Figure 1-figure supplement 3.pdf]

Figure 1-figure supplement 7

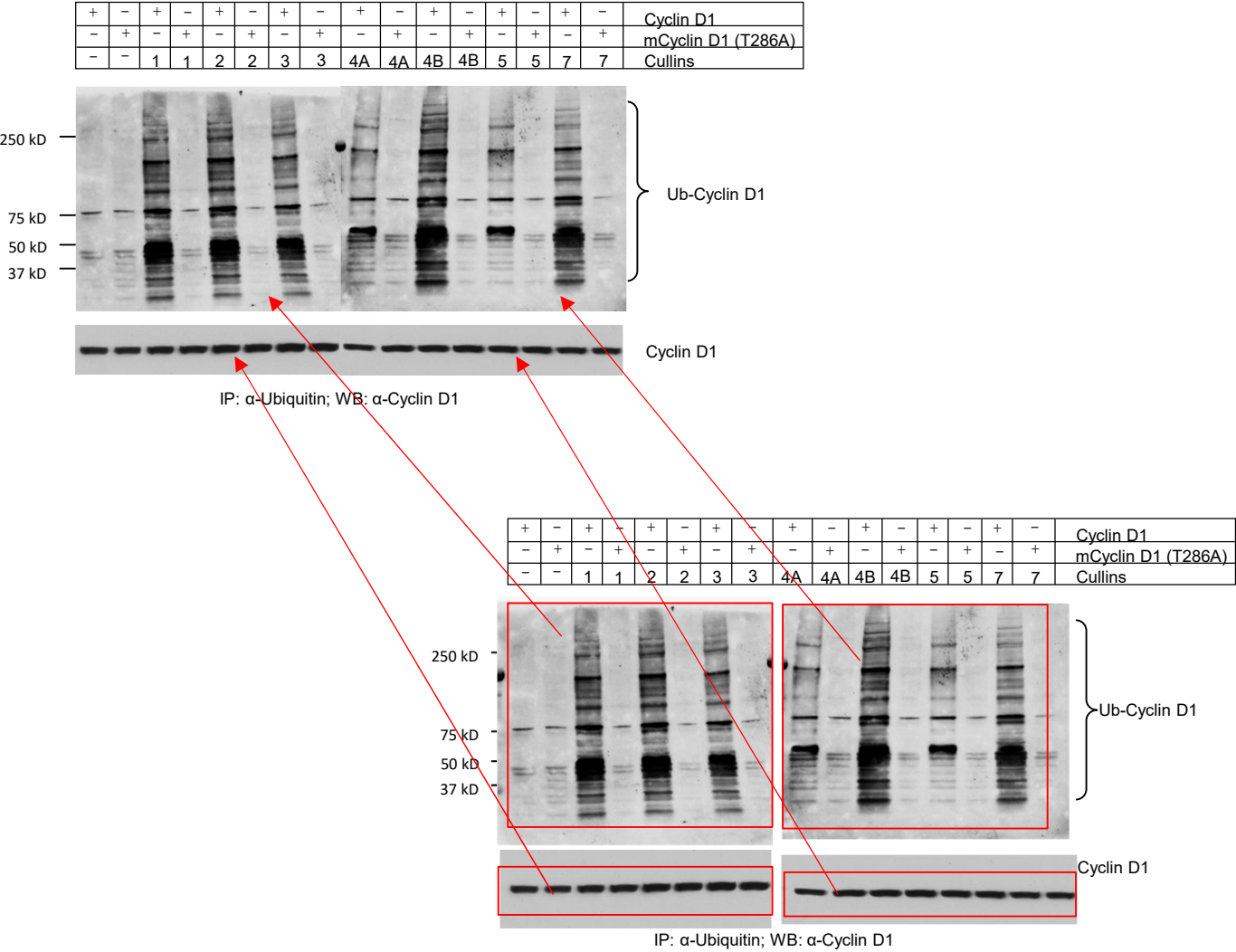

Supplement: Figure 1—source data 2. [file elife-80327-fig1-data2.zip › Original western blot files for Figure 1-figure supplement 7.pdf]

Figure 1-figure supplement 8

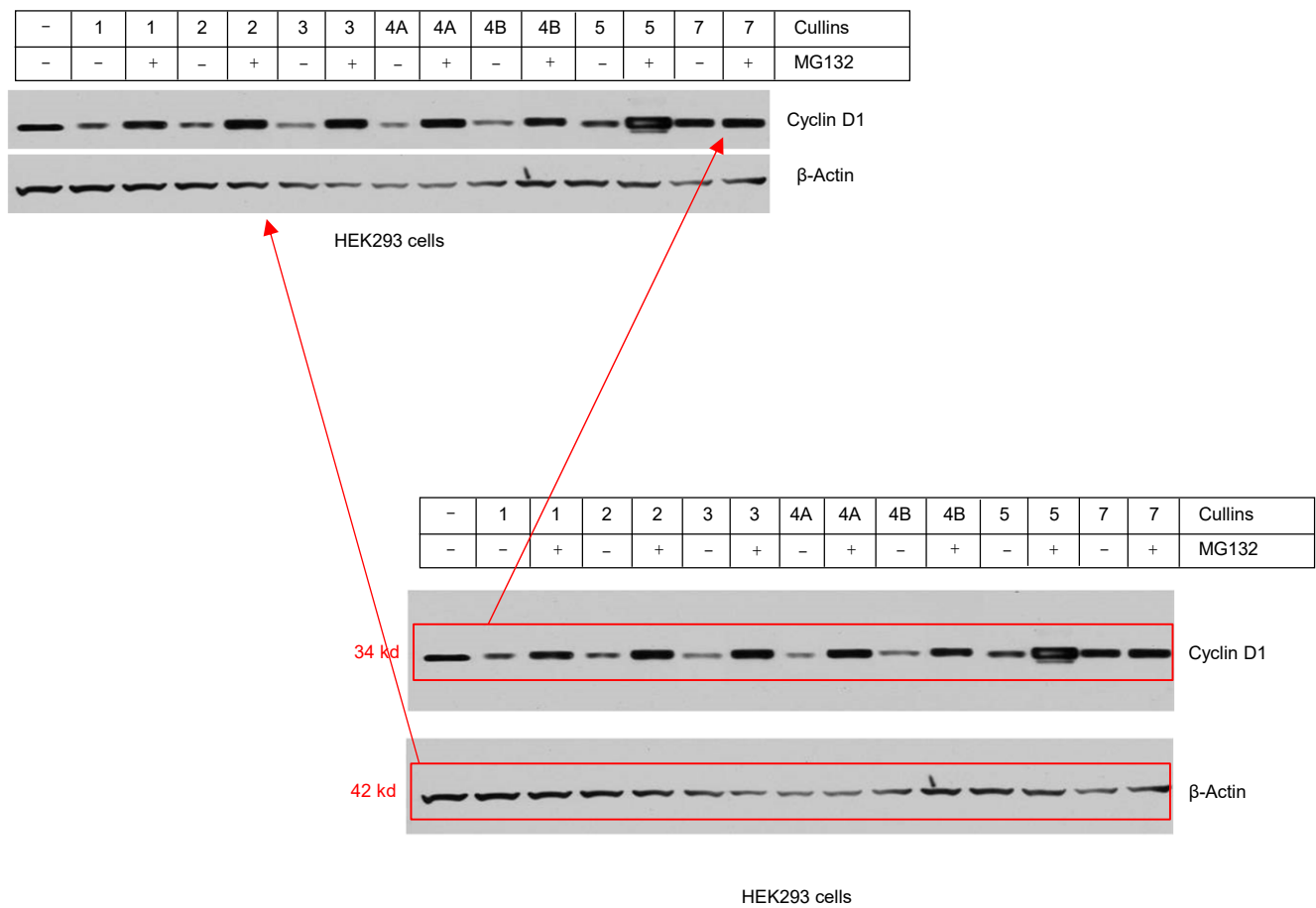

Supplement: Figure 1—source data 2. [file elife-80327-fig1-data2.zip › Original western blot files for Figure 1-figure supplement 8.pdf]

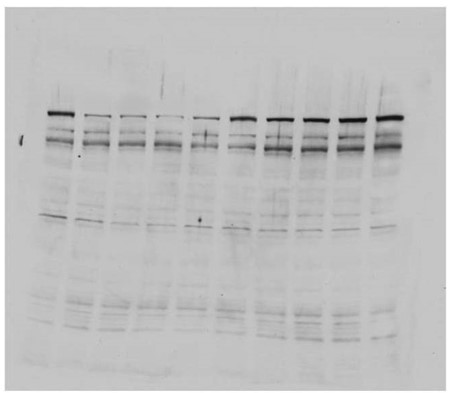

Supplement: Figure 2—source data 2. [file elife-80327-fig2-data2.zip › Figure2-Figure supplement 14-1.jpg]

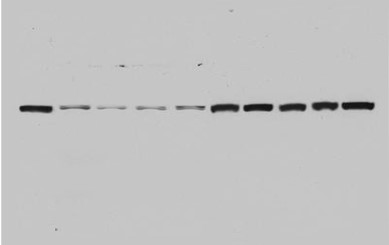

Supplement: Figure 2—source data 2. [file elife-80327-fig2-data2.zip › Figure2-Figure supplement 14-2.jpg]

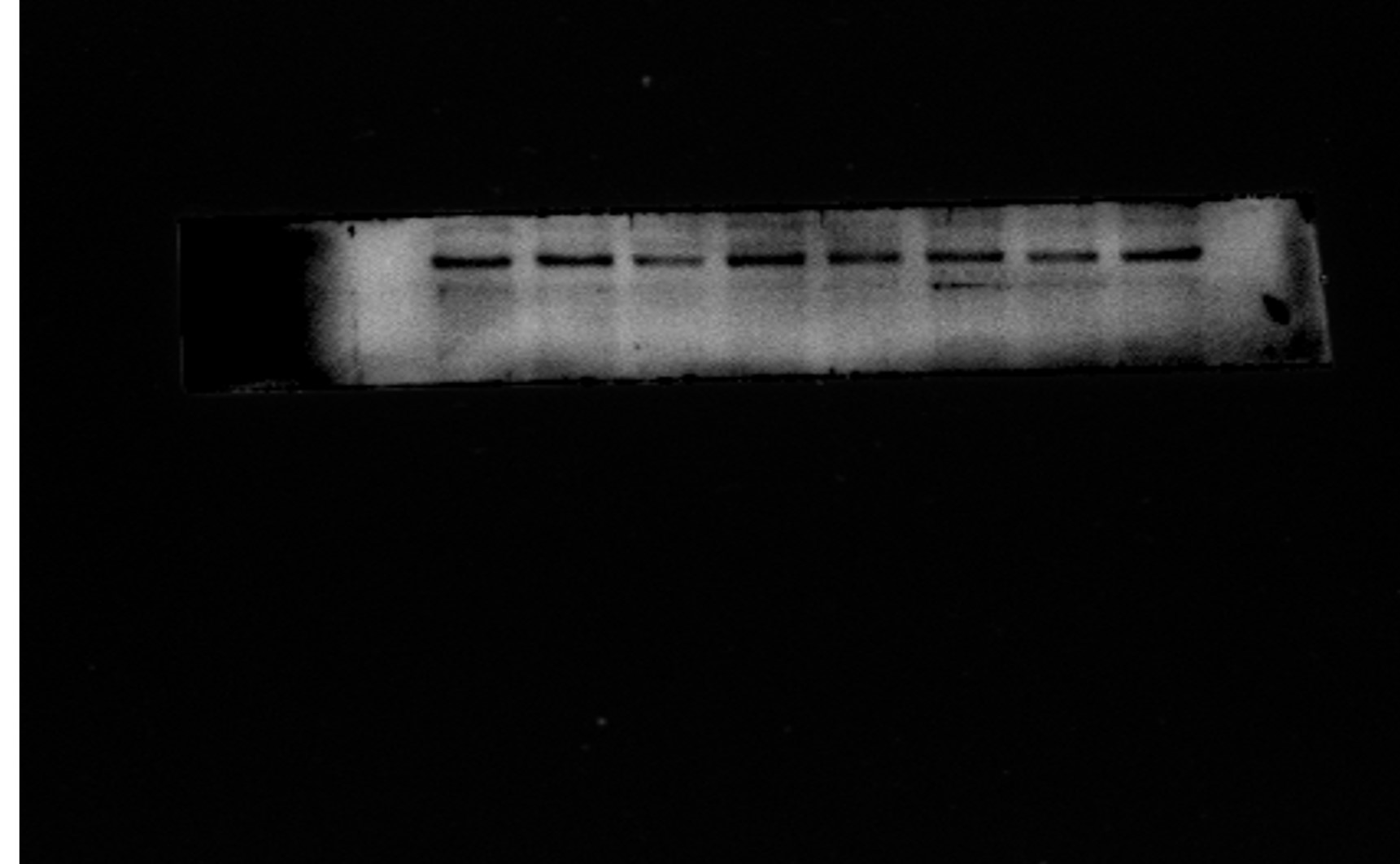

Supplement: Figure 2—source data 2. [file elife-80327-fig2-data2.zip › Figure2-Figure supplement 15-1.jpg]

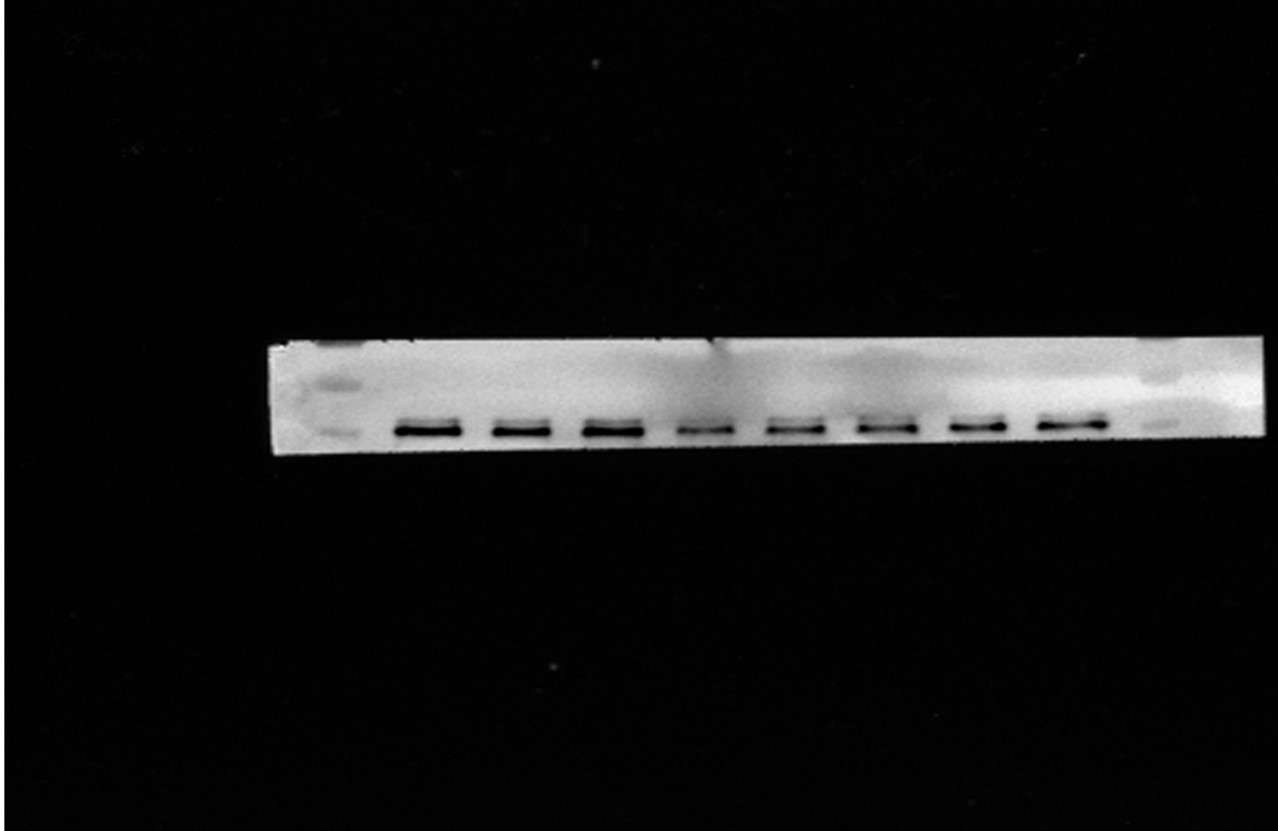

Supplement: Figure 2—source data 2. [file elife-80327-fig2-data2.zip › Figure2-Figure supplement 15-2.jpg]

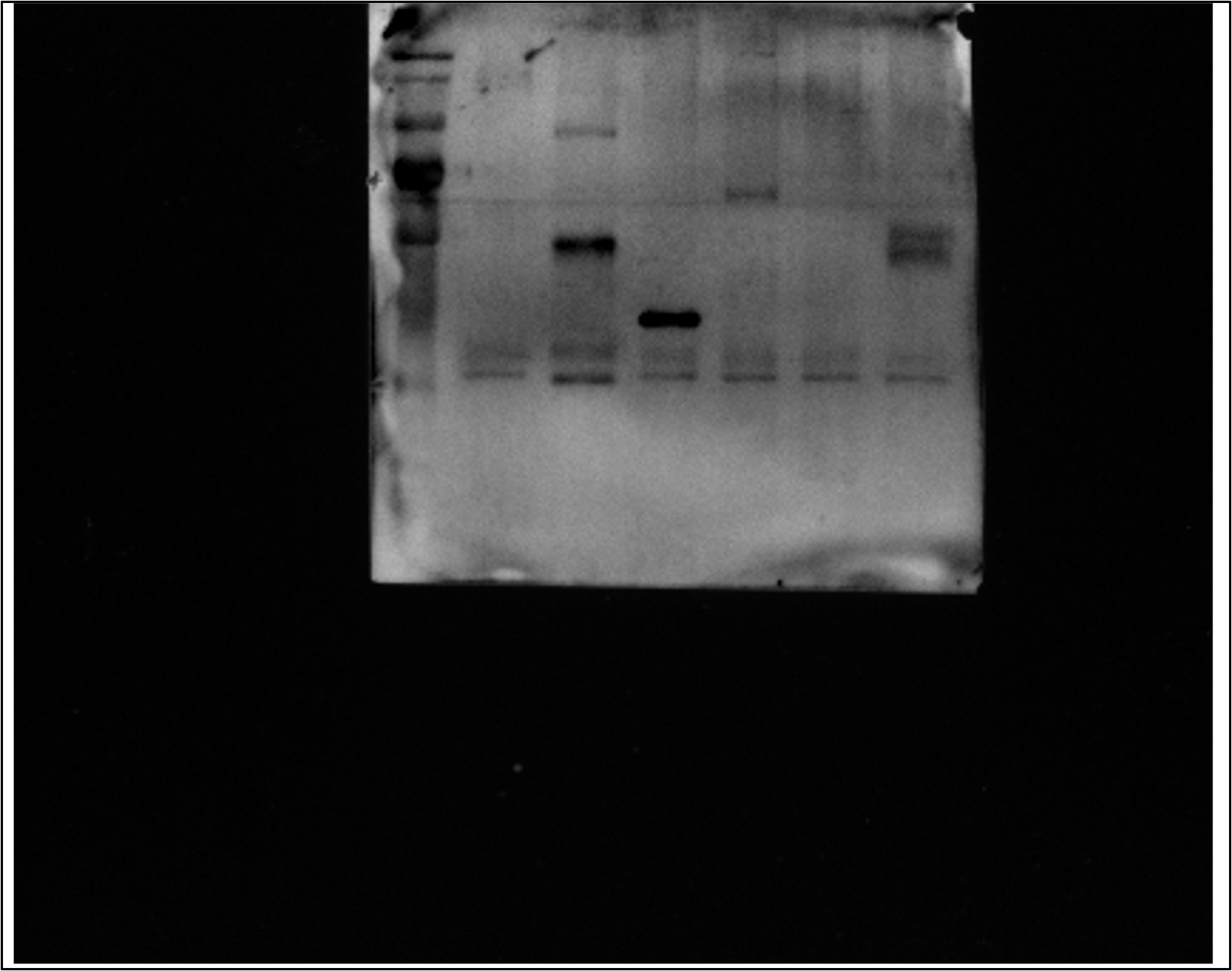

Supplement: Figure 2—source data 2. [file elife-80327-fig2-data2.zip › Figure2-Figure supplement 16.jpg]

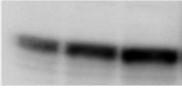

Supplement: Figure 2—source data 2. [file elife-80327-fig2-data2.zip › Figure2A-1.jpg]

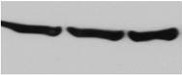

Supplement: Figure 2—source data 2. [file elife-80327-fig2-data2.zip › Figure2A-2.jpg]

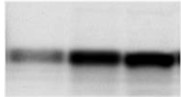

Supplement: Figure 2—source data 2. [file elife-80327-fig2-data2.zip › Figure2D-1.jpg]

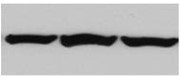

Supplement: Figure 2—source data 2. [file elife-80327-fig2-data2.zip › Figure2D-2.jpg]

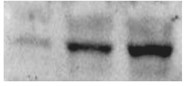

Supplement: Figure 2—source data 2. [file elife-80327-fig2-data2.zip › Figure2G-1.jpg]

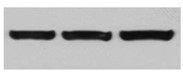

Supplement: Figure 2—source data 2. [file elife-80327-fig2-data2.zip › Figure2G-2.jpg]

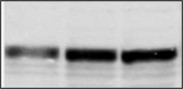

Supplement: Figure 2—source data 2. [file elife-80327-fig2-data2.zip › Figure2J-1.jpg]

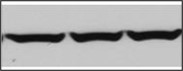

Supplement: Figure 2—source data 2. [file elife-80327-fig2-data2.zip › Figure2J-2.jpg]

Figure 2-figure supplement 4

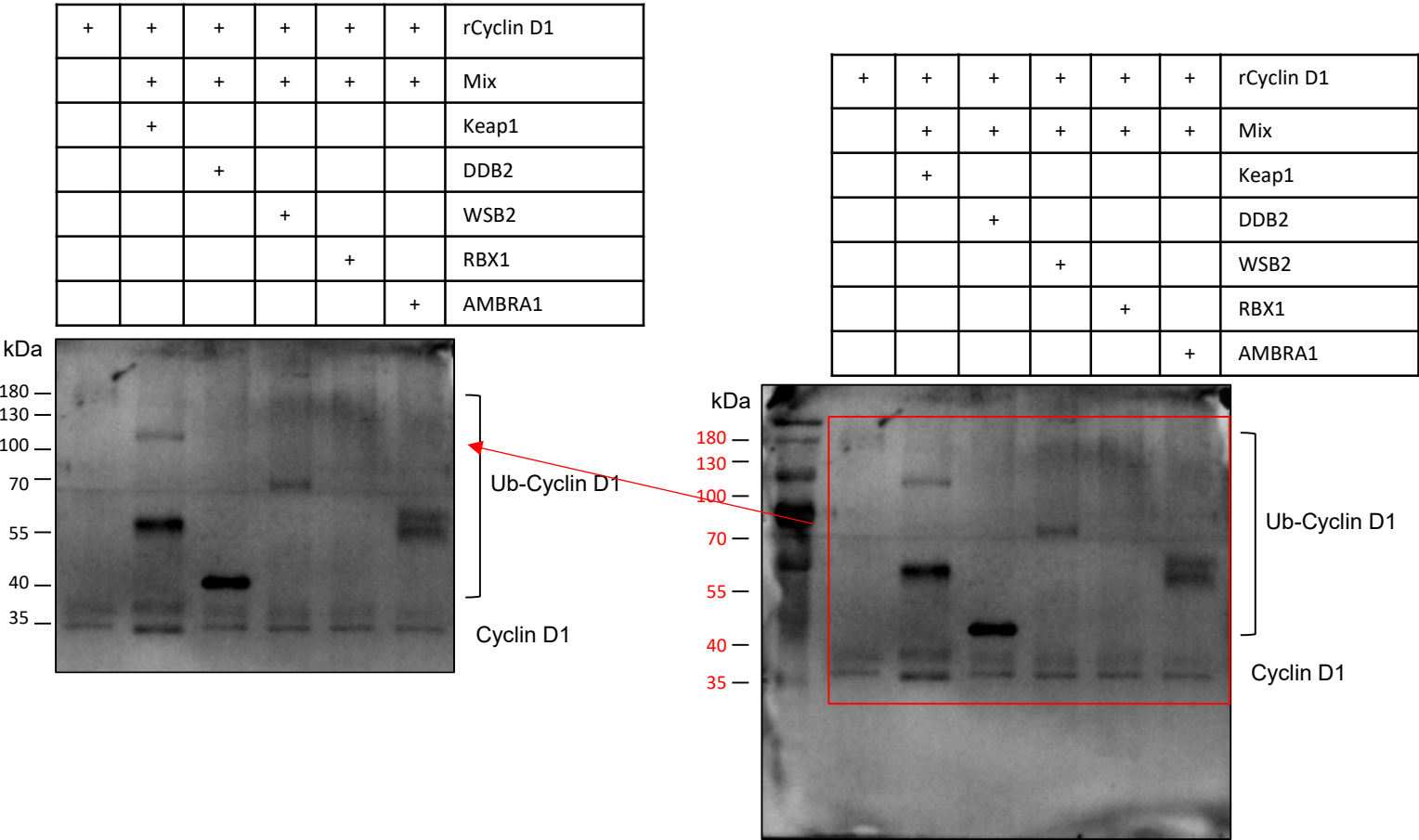

Supplement: Figure 2—source data 2. [file elife-80327-fig2-data2.zip › Original western blot files for Figure 2-figure supplement 4.pdf]

Figure 2A

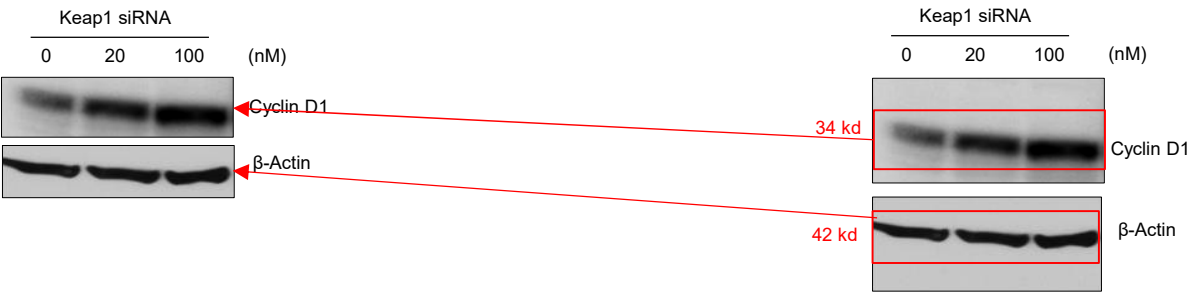

Figure 2D

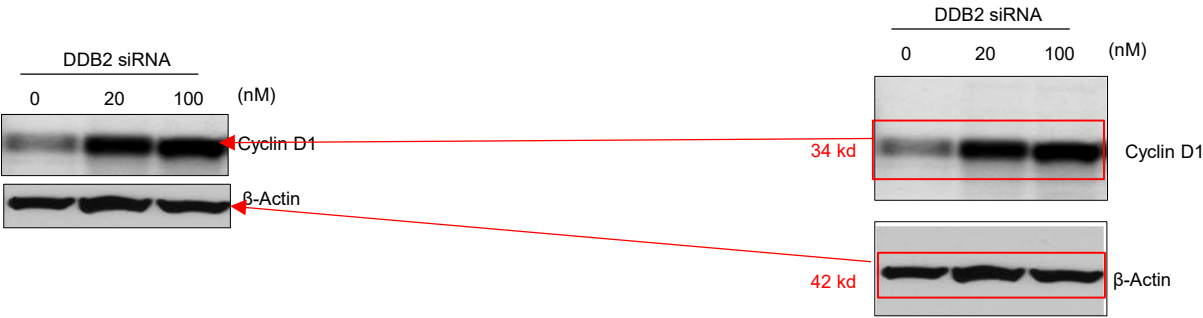

Figure 2G

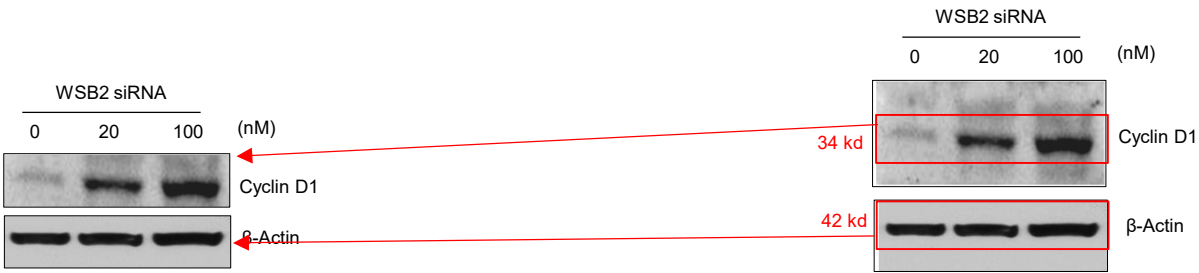

Figure 2J

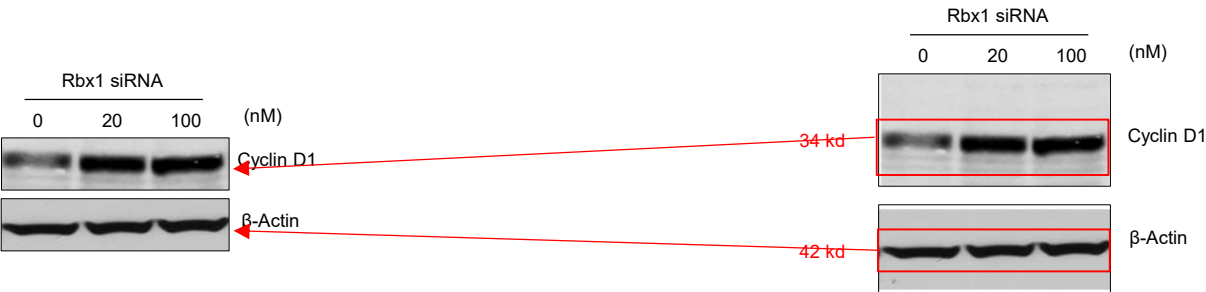

Supplement: Figure 2—source data 2. [file elife-80327-fig2-data2.zip › Original western blot files for Figure 2.pdf]
