# Supplementary material for: Multiple cullin-associated E3 ligases regulate cyclin D1 protein stability
Source: eLife. 2023 Nov 9;12:e80327. doi: 10.7554/eLife.80327 (PMC10651173; doi:10.7554/eLife.80327)

Figure 2-figure supplement 2

+	+	+	+	+	−	−	−	−	−	WT Cyclin D1
−	−	−	−	−	+	+	+	+	+	Cyclin D1 (T286A)
−	+	−	−	−	−	+	−	−	−	Keap1
−	−	+	−	−	−	−	+	−	−	WSB2
−	−	−	+	−	−	−	−	+	−	DDB2
−	−	−	−	+	−	−	−	−	+	Rbx1

+	+	+	+	+	−	−	−	−	−	WT Cyclin D1
−	−	−	−	−	+	+	+	+	+	Cyclin D1 (T286A)
−	+	−	−	−	−	+	−	−	−	Keap1
−	−	+	−	−	−	−	+	−	−	WSB2
−	−	−	+	−	−	−	−	+	−	DDB2
−	−	−	−	+	−	−	−	−	+	Rbx1

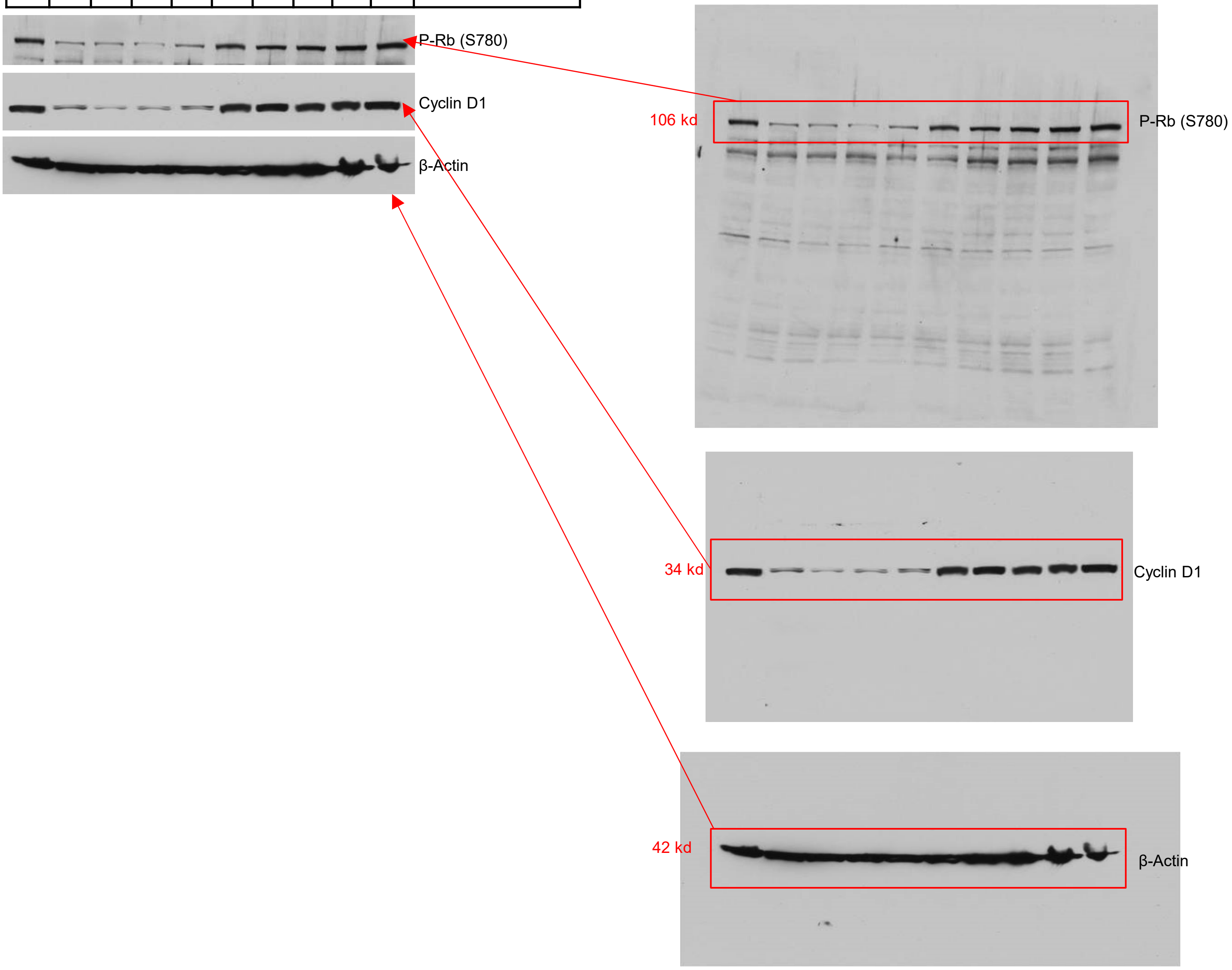

Supplement: Figure 2—source data 2. [file elife-80327-fig2-data2.zip › Original western blot files for Figure 2-figure supplement 2.pdf]
