# Supplementary material for: Multiple cullin-associated E3 ligases regulate cyclin D1 protein stability
Source: eLife. 2023 Nov 9;12:e80327. doi: 10.7554/eLife.80327 (PMC10651173; doi:10.7554/eLife.80327)

Figure 2-figure supplement 3

+	-	+	-	+	-	+	-	WT Cyclin D1
-	+	-	+	-	+	-	+	Cyclin D1 (K269R)
-	-	+	+	-	-	-	-	Keap1
-	-	-	-	+	+	-	-	DDB2
-	-	-	-	-	-	+	+	AMBAR1

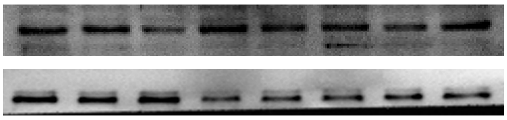

+	-	+	-	+	-	+	-	WT Cyclin D1
-	+	-	+	-	+	-	+	Cyclin D1 (K269R)
-	-	+	+	-	-	-	-	Keap1
-	-	-	-	+	+	-	-	DDB2
-	-	-	-	-	-	+	+	AMBAR1

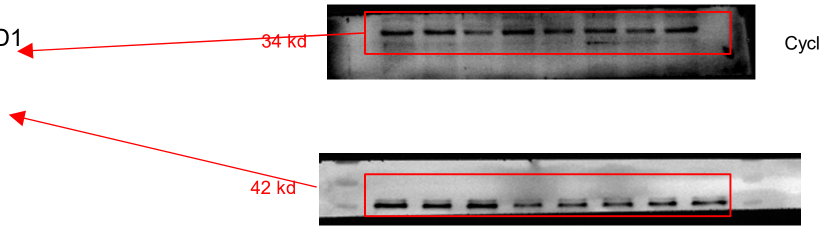

Supplement: Figure 2—source data 2. [file elife-80327-fig2-data2.zip › Original western blot files for Figure 2-figure supplement 3.pdf]
